# Six host-range restricted poxviruses from three genera induce distinct gene expression profiles in an in vivo mouse model

**DOI:** 10.1186/s12864-015-1659-1

**Published:** 2015-07-08

**Authors:** Kristy Offerman, Armin Deffur, Olivia Carulei, Robert Wilkinson, Nicola Douglass, Anna-Lise Williamson

**Affiliations:** Division of Medical Virology, Department of Clinical Laboratory Sciences, University of Cape Town, Cape Town, South Africa; Clinical Infectious Diseases Research Initiative, University of Cape Town, Cape Town, South Africa; Institute of Infectious Disease and Molecular Medicine, University of Cape Town, Cape Town, South Africa; Department of Medicine, University of Cape Town, Cape Town, South Africa; The Francis Crick Institute Mill Hill Laboratory, London, NW7 1AA UK; Department of Medicine, Imperial College, London, W2 1PG UK; National Health Laboratory Service, Groote Schuur Hospital, Cape Town, South Africa

**Keywords:** Poxvirus, Vaccine, Micro-array, LSDV, MVA, Avipoxviruses

## Abstract

**Background:**

Host-range restricted poxviruses make promising vaccine vectors due to their safety profile and immunogenicity. An understanding of the host innate immune responses produced by different poxvirus vectors would aid in the assessment, selection and rational design of improved vaccines for human and veterinary applications. Novel avipoxviruses are being assessed to determine if they are different from other poxvirus vectors. Analysis of the transcriptome induced in a mouse model would aid in determining if there were significant differences between different poxvirus vectors which may reflect different adjuvant potential as well as establish if they should be further evaluated as vaccine vectors.

**Results:**

We compared host transcript abundance in the spleens of BALB/c mice twenty four hours after intravenous infection (10^5^ pfu/mouse) with six host-restricted poxvirus species from three genera, namely Lumpy Skin Disease virus (LSDV), Canarypox virus (CNPV), Fowlpox virus (FWPV), modified vaccinia Ankara (MVA) and two novel South African avipoxviruses, Feral Pigeonpox virus (FeP2) and Penguinpox virus (PEPV). These six viruses produced qualitatively and quantitatively distinct host responses with LSDV, followed by MVA, inducing the greatest interferon (IFN) response. FeP2 and PEPV caused very little change to host transcript abundance compared to the other 4 viruses tested. CNPV and FWPV induced the up regulation of two immunoglobulin genes (Ighg and Ighg3 (IgG3)) with CNPV inducing a third, Ighm (IgM). HIV-1–specific IgG3 antibodies have been correlated with decreased risk of HIV-1 infection in the RV144 trial, which included a CNPV-based vector (Yates et al. (Sci Transl Med, 6(228) p228, 2014). Up regulation of IgG3 by CNPV and FWPV but not the other poxviruses tested in vivo, implies that these two avipoxvirus-vector backbones may be involved in stimulation of the clinically important IgG3 antibody subclass. Differential transcript abundance associated with the different poxviruses is further discussed with particular emphasis on responses related to immune responses.

**Conclusion:**

Six, genetically diverse host-restricted poxviruses produce different responses in a mouse model early after infection. These differences may affect the immune response induced to vaccine antigen in vectors based on these viruses. The two novel avipoxviruses were clearly distinguishable from the other viruses.

**Electronic supplementary material:**

The online version of this article (doi:10.1186/s12864-015-1659-1) contains supplementary material, which is available to authorized users.

## Background

Although a number of different poxvirus-based vaccine vectors are available [1–7], there is still a need for additional vaccine vectors as well as improvement of the vectors. The unique response elicited by the host to different vectors means that vectors can be selected or engineered according to a desired host response. Host-range restricted poxviruses have been shown to successfully activate the host immune system [8, 9] and evidence exists that each virus does this in a different way, with an accompanying different pattern of transcript abundance [10–15]. The poxviruses ALVAC (based on canarypox virus), modified vaccinia Ankara (MVA) and NYVAC (both based on vaccinia virus (VACV) and have specific deletions) produce distinct innate immune profiles, characterised by different induction of pro-inflammatory and antiviral cytokines and chemokines in both rhesus monkeys and human PBMC [16].

It has been shown that in non-permissive cells, Fowlpox virus (FWPV) proceeds further into the poxvirus life cycle than Canarypox virus (CNPV) [17]. Heterologous HIV gag/pol and env genes are more efficiently expressed by FWPV than CNPV in vitro due to longer transgene expression [18]. However, the only successful HIV-1 vaccine clinical trial to date (31.2 % protection from HIV-1 infection) has been the Thai RV144 trial involving priming with ALVAC expressing HIV-1 gp120/Gag-Pro and boosting with a recombinant glycoprotein 120 subunit, AIDSVAX [7]. Head to head comparisons of poxvirus-vectored vaccines would help to establish the differences between the different vaccine vectors and the vaccine-induced response to achieve protection against pathogens and cancers.

Innate immunity is critical for directing the adaptive immune response to antigen and influences the magnitude and quality of the long-lived, protective immune responses to pathogens or vaccines [19]. Application of the systems biology approach to vaccine development (“systems vaccinology”) and establishment of innate immune signatures has proven useful in predicting the immunogenicity of the highly effective yellow fever vaccine (YF-17D) [20], seasonal influenza vaccines [21] and the immunogenic but inefficacious Merck Adenovirus type 5 (Ad5) based HIV vaccine [22]. A better understanding of the mechanisms underlying the optimal innate immune responses would aid rational vaccine development.

Type 1 interferons (IFNα/β) are expressed rapidly in response to viral infection, and, in turn activate many interferon stimulated genes (ISGs) which exert various antiviral effector functions. A fine balance of IFN is required for successful vaccination using a live virus vector. The vector should induce enough type I IFNs to activate the immune system, yet not enough to inhibit viral DNA replication and gene expression before antigen presentation can occur [23]. This is corroborated by Johnson et al. (2012), who compared recombinant (r) Ad types 5, 28 and 35. Specific IFN-α induction by rAd28 and rAd35 significantly lowered the immunogenicity of these vectors compared to rAd5 which did not induce IFN-α expression [24]. The effect of type 1 IFN responses on different vaccines requires delineation of innate immune signatures and how they determine subsequent adaptive responses.

Microarray analyses performed in vitro have been used to investigate the effects of VACV ([12], MVA [11] and NYVAC [13] infection on HeLa cell gene expression. Gene expression profiles in human monocyte derived dendritic cells (MDDCs) have also been generated with MVA, NYVAC [14] and ALVAC [15]. Furthermore, a comparison of the closely related VACV-derived vectors NYVAC and MVA revealed significant differences in antigen production and host gene dysregulation in cell culture [25]. Consequently we hypothesized that genetically diverse poxvirus strains would induce significant differences in host gene expression. The interaction of poxviruses with the host is not just dependent on the actual cell infected by the virus but also on the factors secreted by those infected cells and their effects on the surrounding cells. Although in vitro expression studies have provided useful information, gene expression profiles performed in cell culture may not accurately reflect the changes in the system that occurs as a result of infection in vivo. A recent study in Rhesus Macaques showed that ALVAC induced distinct cytokine and chemokine levels compared to the vaccinia virus-based vectors MVA and NYVAC and that multiple subsets of peripheral blood mononuclear cells (PBMC) are likely to contribute to the overall response to different poxviruses [16].

In this study we compared the effects of the capripoxvirus, lumpy skin disease virus (LSDV), the orthopoxvirus, MVA, and the avipoxviruses, CNPV, FWPV, a novel pigeonpox virus (FeP2) [26, 27] and a novel penguinpox virus (PEPV) [27–29] on host gene expression profiles in the spleens of BALB/c mice. None of these viruses complete their replication cycle in mice.

## Results

### Comparison of the host responses to different poxviruses

We compared the differential host gene expression induced by six host-restricted poxviruses, MVA, LSDV, FWPV, CNPV, FeP2 and PEPV, in the spleens of BALB/c mice 24 h post infection. Transcripts with an adjusted *p*-value < 0.05 were described as up-regulated if they had a log_2_ fold change (FC) of ≥1, or down-regulated if they had a log_2_FC of ≤ −1. A summary of the number of up and down-regulated genes is given in Table 1. Full gene lists are given in supplementary data (Additional file 1). Quantitative RT-PCR showed all three housekeeping genes (GAPDH, HPRT and CD51) to be expressed at similar levels to those of the PBS control for all samples. Both IRF7 and Zbp1 were upregulated by all poxviruses tested. IGFbp3 was shown to be downregulated by all viruses. Overall the quantitative RT-PCR was more sensitive than the microarray, but the trend observed in up- and down-regulation of host gene expression was similar for qRT-PCR and microarray analysis.

**Table 1:** Summary of the number of significantly up- and down-regulated transcripts with adjusted *p*-value < 0.05

	Up-regulated Log_2_ FC > 1	Down-regulated Log_2_ FC < −1
MVA	299 (42NA)	177 (86NA)
LSDV	463 (111NA)	85 (11NA)
FWPV	433 (101NA)	62 (28NA)
CNPV	280 (31NA)	47 (11NA)
FeP2	20 (1NA)	3 (0NA)
PEPV	68 (6NA)	19 (2NA)

Genes are described as upregulated if they had a fold change of ≥2, or down-regulated if they had a Log_2_ Fold change of ≤ −1. These included genes that are not annotated and therefore do not have an Entrez ID. The number of genes without annotation are indicated in brackets

**Fig. 1:**
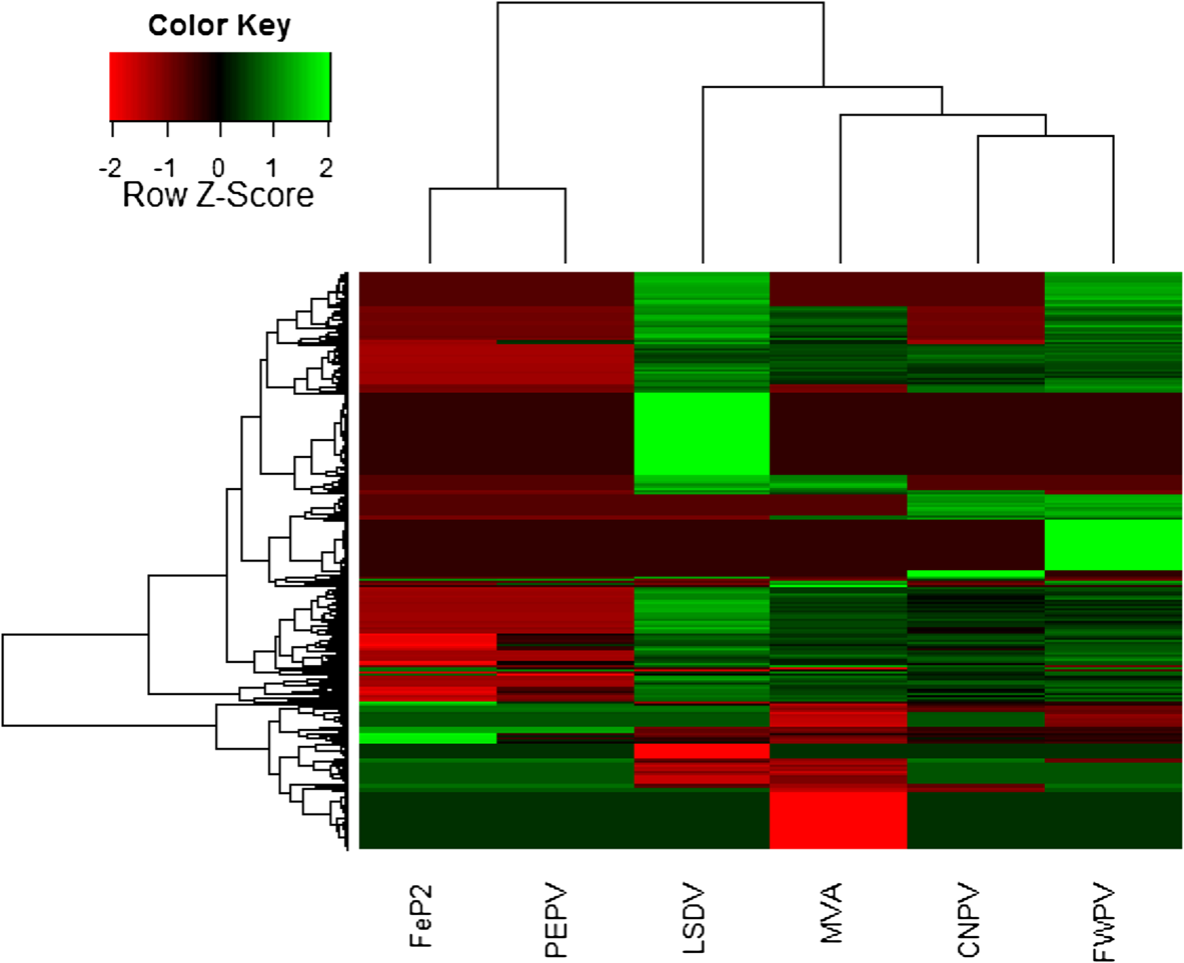
Heatmap comparing the differential expression induced in mouse spleens in response to pigeonpox (FeP2), penguinpox (PEPV), lumpy skin disease virus (LSDV), modified vaccinia Ankara (MVA), canarypox virus (CNPV) and fowlpox virus (FWPV). Only genes (with *p*-value < 0.05) with log_2_ fold change induction above or below the cutoff (±1) as compared to the mock infected control are shown. Unsupervised hierarchical clustering of the samples is represented by dendograms. Clustering analysis and heatmap was performed in the R package, gplots (Warnes, 2009)

**Fig. 2:**
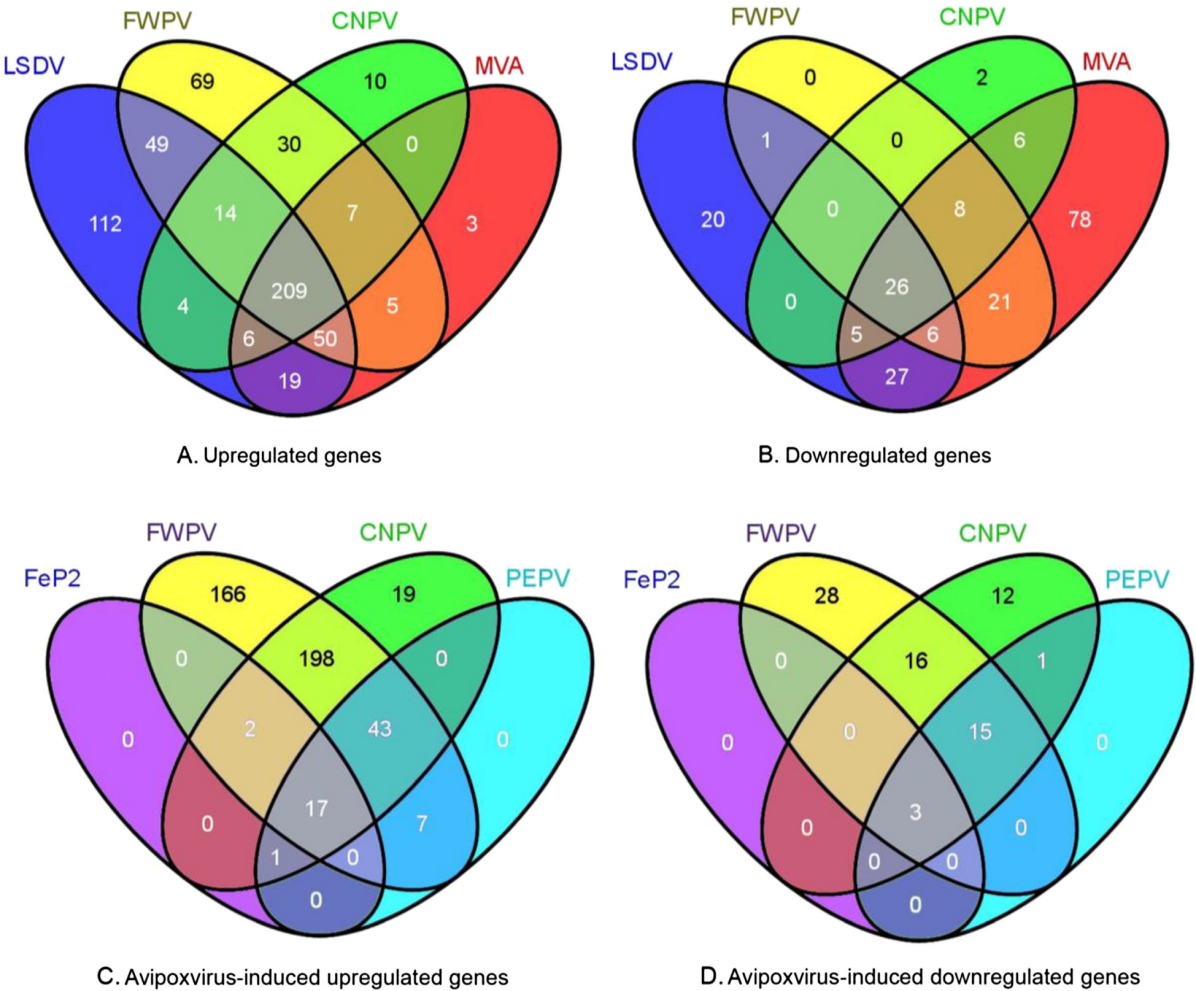
Venn diagrams showing the overlap between the differentially up-regulated (**a**) and down-regulated (**b**) transcripts induced by canarypox virus (CNPV), fowlpox virus (FWPV), modified vaccinia Ankara (MVA) and lumpy skin disease virus (LSDV) and the up-regulated (**c**) and down-regulated (**d**) transcripts induced by the four avipoxviruses. For each diagram, the circles represent the number of differently expressed transcripts regulated by each virus (*p* value ≤ 0.05, log_2_ fold change of ≥ ±1). The numbers in the intersections of each circle represents the number of transcripts common to the respective virus/es

Unsupervised hierarchical clustering based on the genes with *p*-value < 0.05 and log_2_FC above or below cutoff (>1, <−1) showed that each virus induced a unique overall response (Fig. 1). Venn diagrams highlight the number of differences and similarities in the up- and down-regulated genes between the viruses (Fig. 2). Fig. 2 a and b show the differences in transcripts up (A) and (B) down-regulated respectively between FWPV, CNPV, MVA and LSDV. The Venn diagrams comparing FWPV, CNPV, MVA and LSDV indicate that the majority of up-regulated genes are shared amongst these 4 viruses (Fig. 2). The down-regulated genes however, appear largely unique, especially for LSDV and MVA (Fig. 2). FWPV and CNPV down regulate a smaller number of genes in comparison to LSDV and MVA. Comparison of avipoxvirus-induced up- and down-regulated genes shows that FeP2 and PEPV induce significantly less change in host transcript abundance than FWPV and CNPV (Fig. 2). FeP2 induced the lowest response (Fig. 2, Table 1). For all six viruses, more genes were up-regulated than down-regulated (Table 1).

#### Histone transcripts

Previous studies have found that increased detection of histone genes by poxvirus infection is described as an experimental artefact due to the de novo polyadenylation of transcripts by the viral poly-A polymerase [30, 31]. Several histone transcripts (39 in total) were down-regulated in response to virus infection and, because any interpretation of these transcripts would be speculative, these have been excluded from further analysis.

### Immunity and host defence response-related genes

#### Up-regulated immune response genes

Selected up-regulated genes involved in the immune response are listed in Table 2. (Full list of up-regulated genes is given in Additional file 1). Seventeen of these genes are uniquely up-regulated by LSDV. RIG-I (Ddx58) senses viral nucleic acid [32], Cebpb is important for macrophage function [33] and control of inflammatory responses [34], Tap1 and Tap2 genes are involved in antigen presentation to MHC class 1 molecules [35], Ifitm3 and Ifi203 are interferon responsive genes (ISG), c-Myc and Mif are transcription factors and Adar is an RNA editing enzyme. There are eight genes induced by CNPV, FWPV and MVA, which were not up-regulated in LSDV-infected mice. Two of these include the cytidine deaminase, Apobec1, which can edit viral nucleic acid and can thereby limit viral replication [36], and Caspase 1 (Casp1), which is associated with pyroptosis (Table 2).

**Table 2:** Selection of up-regulated genes in mouse spleens in response to MVA, LSDV, CNPV, FWPV, PEPV and FeP2. Differences in Log_2_ Fold Changes (between each virus and the control) are depicted.

Symbol	Name	Entrez	MVA	LSDV	CNPV	FWPV	PEPV	FeP2
**Genes induced by LSDV alone**								
*Oas1b*	*2′-5′ oligoadenylate synthetase 1B*	*23961*	*-*	*1.6*	*-*	*-*	*-*	*-*
*Adar*	*adenosine deaminase, RNA-specific*	*56417*	*-*	*1*	-	-	-	-
*Cebpb*	*CCAAT/enhancer binding protein (C/EBP), beta*	*12608*	*-*	*1*	*-*	*-*	*-*	*-*
*Ddx58 (RIG-1)*	*DEAD (Asp-Glu-Ala-Asp) box polypeptide 58*	*230073*	*-*	*1.3*	*-*	*-*	*-*	*-*
*Grn*	*granulin*	*14824*	*-*	*1.1*	*-*	*-*	*-*	*-*
*Gvin1*	*GTPase, very large interferon inducible 1*	*74558*	*-*	*1.5*	*-*	*-*	*-*	*-*
*Gm17757*	*GTPase, very large interferon inducible 1 pseudogene*	*100417829*	*-*	*1.5*	*-*	*-*	*-*	*-*
*H2-T24*	*histocompatibility 2, T region locus 24*	*15042*	*-*	*1.2*	*-*	*-*	*-*	*-*
*Ifi203*	*interferon activated gene 203*	*15950*	*-*	*1.1*	*-*	*-*	*-*	*-*
*Ifitm3*	*interferon induced transmembrane protein 3*	*66141*	*-*	*1.4*	*-*	*-*	*-*	*-*
*Ifi27l2a*	*interferon, alpha-inducible protein 27 like 2A*	*76933*	*-*	*1.9*	*-*	*-*	*-*	*-*
*Ly6i*	*lymphocyte antigen 6 complex, locus I*	*57248*	*-*	*1*	*-*	*-*	*-*	*-*
*Mif*	*macrophage migration inhibitory factor*	*17319*	*-*	*1.1*	*-*	*-*	*-*	*-*
*Myc*	*myelocytomatosis oncogene*	*17869*	*-*	*1.1*	*-*	*-*	*-*	*-*
*Nlrc5*	*NLR family, CARD domain containing 5*	*434341*	*-*	*1.8*	*-*	*-*	*-*	*-*
*Slfn2*	*schlafen 2*	*20556*	*-*	*1.1*	*-*	*-*	*-*	*-*
Stat1	signal transducer and activator of transcription 1	20846	-	1.4	-	-	-	-
*Tap1*	*transporter 1, ATP-binding cassette, sub-family B (MDR/TAP)*	*21354*	*-*	*1.1*	*-*	*-*	*-*	*-*
*Tap2*	*transporter 2, ATP-binding cassette, sub-family B (MDR/TAP)*	*21355*	*-*	*1*	*-*	*-*	*-*	*-*
*Trim25*	*tripartite motif-containing 25*	*217069*	*-*	*1*	*-*	*-*	*-*	*-*
*Trim34b*	*tripartite motif-containing 34B*	*434218*	*-*	*1.1*	*-*	*-*	*-*	*-*
**Genes induced by CNPV, FWPV and MVA only, and not LSDV.**								
*Casp1*	*caspase 1*	*12362*	*1.1*	*-*	*1.2*	*1.1*	*-*	*-*
*Clec4a2*	*C-type lectin domain family 4, member a2*	*26888*	*1.2*	*-*	*1.5*	*1.1*	-	-
*Ifi205*	*interferon activated gene 205*	*226695*	*1.4*	*-*	*1.6*	*1.8*	*-*	*-*
*Prdx1*	*peroxiredoxin 1*	*18477*	*1*	*-*	*1*	*1.2*	*-*	*-*
*Pnpt1*	*polyribonucleotide nucleotidyltransferase 1*	*71701*	*1.1*	*-*	*1.1*	*1.4*	*-*	*-*
*Scimp*	*SLP adaptor and CSK interacting membrane protein*	*327957*	*1.2*	*-*	*1.2*	*1.5*	*-*	*-*
**Genes induced by avipoxviruses only (CNPV, FWPV, FEP2 and/ or PEPV)**								
**Anxa1**	**annexin A1**	**16952**	**-**	**-**	**1.6**	**1.8**	**-**	**-**
**Apobec1**	**apolipoprotein B mRNA editing enzyme, catalytic polypeptide 1**	**11810**	**-**	**-**	**1.2**	**1.2**	**-**	**-**
**Ccl6**	**chemokine (C-C motif) ligand 6**	**20305**	**-**	**-**	**1.3**	**1.3**	**-**	**-**
**Ear2**	**eosinophil-associated, ribonuclease A family, member 2**	**13587**	**-**	**-**	**1.3**	**1.5**	**-**	**-**
**Hsbp1**	**heat shock factor binding protein 1**	**68196**	**-**	**-**	**1.2**	**1.2**	**-**	**-**
**Ighg**	**Immunoglobulin heavy chain (gamma polypeptide)**	**380794**	**-**	**-**	**1.5**	**1.6**	**-**	**-**
**Ighg3**	**Immunoglobulin heavy constant gamma 3**	**380795**	**-**	**-**	**1.3**	**1.2**	**-**	**-**
**Lilrb3**	**leukocyte immunoglobulin-like receptor, subfamily B (with TM and ITIM domains), member 3**	**18733**	**-**	**-**	**1.1**	**1**	**-**	**-**
**Marco**	**macrophage receptor with collagenous structure**	**17167**	**-**	**-**	**1**	**-**	**1.5**	**1.5**
**Pf4**	**platelet factor 4**	**56744**	**-**	**-**	**1.2**	**1.1**	**-**	**-**
**Pram1**	**PML-RAR alpha-regulated adaptor molecule 1**	**378460**	**-**	**-**	**1.1**	**1.1**	**-**	**-**
**Psma1**	**proteasome (prosome, macropain) subunit, alpha type 1**	**26440**	**-**	**-**	**1.1**	**1.3**	**-**	**-**
**Genes induced by FWPV only.**								
***Aif1***	***allograft inflammatory factor 1***	***11629***	***-***	***-***	***-***	***1.1***	***-***	***-***
***Anxa2***	***annexin A2***	***12306***	***-***	***-***	***-***	***1.1***	***-***	***-***
***Ddx18***	***DEAD (Asp-Glu-Ala-Asp) box polypeptide 18***	***66942***	***-***	***-***	***-***	***1.1***	***-***	***-***
***Dcn***	***decorin***	***13179***	***-***	***-***	***-***	***1.5***	***-***	***-***
***Fgl2***	***fibrinogen-like protein 2***	***14190***	***-***	***-***	***-***	***1.2***	***-***	***-***
***Gsdmd***	***gasdermin D***	***69146***	***-***	***-***	***-***	***1.2***	***-***	***-***
***Myd88***	***myeloid differentiation primary response gene 88***	***17874***	***-***	***-***	***-***	***1.2***	***-***	***-***
***Nos2***	***nitric oxide synthase 2, inducible***	***18126***	***-***	***-***	***-***	***1.1***	***-***	***-***
***Nod1***	***nucleotide-binding oligomerization domain containing 1***	***107607***	***-***	***-***	***-***	***1***	***-***	***-***
***Pdcd5***	***programmed cell death 5***	***56330***	***-***	***-***	***-***	***1.2***	***-***	***-***
***Psmc6***	***proteasome (prosome, macropain) 26S subunit, ATPase, 6***	***67089***	***-***	***-***	***-***	***1***	***-***	***-***
***Prmt1***	***protein arginine N-methyltransferase 1***	***15469***	***-***	***-***	***-***	***1***	***-***	***-***
***Serpinb6b***	***serine (or cysteine) peptidase inhibitor, clade B, member 6b***	***20708***	***-***	***-***	***-***	***1.2***	***-***	***-***
**Genes induced by CNPV only.**								
**Ctsl**	**cathepsin L**	**13039**	**-**	**-**	**1.1**	**-**	**-**	**-**
**Ighm**	**immunoglobulin heavy constant mu**	**16019**	**-**	**-**	**1.1**	**-**	**-**	**-**
**Ly96**	**lymphocyte antigen 96**	**17087**	**-**	**-**	**1.2**	**-**	**-**	**-**
**Pomp**	**proteasome maturation protein**	**66537**	**-**	**-**	**1**	**-**	**-**	**-**
**Genes induced by LSDV AND MVA, but not by the AVIPOXVIRUSES**								
Hsh2d	hematopoietic SH2 domain containing	209488	1.1	1.4	-	-	-	-
Mov10	Moloney leukemia virus 10	17454	1.1	1.5	-	-	-	-
Parp11	poly (ADP-ribose) polymerase family, member 11	101187	1	1.4	-	-	-	-
Slfn8	schlafen 8	276950	1.2	1.5	-	-	-	-
**Other**								
Oas1a	2′-5′ oligoadenylate synthetase 1A	246730	1.5	2.7	1.6	1.4	-	-
Oas1g	2′-5′ oligoadenylate synthetase 1G	23960	2.3	3.9	2.5	2.3	-	-
Oas2	2′-5′ oligoadenylate synthetase 2	246728	2.1	3.4	2	1.6	-	-
Oas3	2′-5′ oligoadenylate synthetase 3	246727	1.1	2.6	1.3	1	-	-
Oasl1	2′-5′ oligoadenylate synthetase-like 1	231655	2.6	3.5	2.4	2.5	-	-
Oasl2	2′-5′ oligoadenylate synthetase-like 2	23962	2	3.4	2	2	-	-
Amica1	adhesion molecule, interacts with CXADR antigen 1	270152	-	1.1	-	1.1	-	-
Angptl4	angiopoietin-like 4	57875	1.1	1.5	-	1.6	1.1	-
Asb13	ankyrin repeat and SOCS box-containing 13	142688	1.2	1	1.1	1.3	-	-
Anxa4	annexin A4	11746	1.8	1.9	1.7	2.1	1.1	-
Apol9b	apolipoprotein L 9b	71898	2.3	2.7	2.6	2.4	-	-
Bst2	bone marrow stromal cell antigen 2	69550	2.3	3	2.1	2.2	-	-
Casp4	caspase 4, apoptosis-related cysteine peptidase	12363	1.9	1.6	1.9	2	-	-
Ctsc	cathepsin C	13032	1.1	1.1	-	1.1	-	-
Cd274	CD274 antigen	60533	1.9	2.3	1.6	2.1	1.2	-
Cd5l	CD5 antigen-like	11801	1.2	1.5	1.4	1.1	-	-
Cd69	CD69 antigen	12515	1.8	1.8	1.5	1.7	-	-
Ccl2 (MCP1)	chemokine (C-C motif) ligand 2	20296	3.5	3.3	2.9	3.3	2.8	-
Ccl3 (MIP-1α)	chemokine (C-C motif) ligand 3	20302	2	2.1	2.3	2	1.4	-
Ccl7	chemokine (C-C motif) ligand 7	20306	3	2.9	2.6	2.9	2.7	1.5
Ccr5	chemokine (C-C motif) receptor 5	12774	1.1	1.5	1.3	1.3	-	-
Ccrl2	chemokine (C-C motif) receptor-like 2	54199	-	1.5	1.2	1.4	-	-
Cxcl10 (IP-10)	chemokine (C-X-C motif) ligand 10	15945	2.7	3	2.2	2.8	1.7	-
Cxcl11 (I-TAC)	chemokine (C-X-C motif) ligand 11	56066	4.5	4.4	3.4	4.3	1.5	-
Cxcl9 (MIG)	chemokine (C-X-C motif) ligand 9	17329	2.1	2.3	-	1.8	-	-
Chi3l3	chitinase 3-like 3	12655	-	1.2	1.4	1.6	-	-
Csf2rb2	colony stimulating factor 2 receptor, beta 2, low-affinity (granulocyte-macrophage)	12984	-	1.5	-	1.7	-	-
C1qa	complement component 1, q subcomponent, alpha polypeptide	12259	-	1.1	1	-	-	-
C2	complement component 2 (within H-2S)	12263	1.5	1.8	1.7	1.6	1	-
Cfb	complement factor B	14962	2.2	2.6	1.6	2	1	-
Cdkn1a (P21)	cyclin-dependent kinase inhibitor 1A	12575	1.7	2	1.5	2	1.2	-
Cstb	cystatin B	13014	1.4	1	1.5	1.5	-	-
Cst7	cystatin F (leukocystatin)	13011	1.2	1.4	1.1	1.4	-	-
Cmpk2	cytidine monophosphate (UMP-CMP) kinase 2, mitochondrial	22169	1.3	2	1.3	1.3	-	-
Cycs	cytochrome c, somatic	13063	-	1.1	-	1.1	-	-
Ctla2a	cytotoxic T lymphocyte-associated protein 2 alpha	13024	1.1	1	1.1	1.3	-	1.4
Ddx60	DEAD (Asp-Glu-Ala-Asp) box polypeptide 60	234311	1.7	2.6	1.5	1.4	-	-
Dhx58 (LGP2)	DEXH (Asp-Glu-X-His) box polypeptide 58	80861	1.7	2.5	1.6	1.5	-	-
Dram1	DNA-damage regulated autophagy modulator 1	71712	1.2	1.4	-	1.3	-	-
Ddit4	DNA-damage-inducible transcript 4	74747	1.2	1.2	1.8	1.3	1.4	-
Eif2ak2 (PKR)	eukaryotic translation initiation factor 2-alpha kinase 2	19106	1.1	2.1	1.1	1.1	-	-
Daxx	Fas death domain-associated protein	13163	2	2.3	1.7	1.9	-	-
Fcgr1	Fc receptor, IgG, high affinity I	14129	2.4	2.6	2.4	2.6	1.4	-
Fcgr4	Fc receptor, IgG, low affinity IV	246256	2.5	3.6	2.8	2.9	1.8	-
Fpr1	formyl peptide receptor 1	14293	1.3	1.1	1.4	1.4	-	-
Fpr2	formyl peptide receptor 2	14289	1.8	1.5	1.7	1.9	-	-
Glipr2	GLI pathogenesis-related 2	384009	1.4	1.5	1.1	1.5	-	-
Gp49a	glycoprotein 49 A	14727	2.6	2.4	2.7	2.7	1.9	2
Gca	grancalcin	227960	1.2	1.3	1.4	1.6	-	-
Gzma	granzyme A	14938	1.8	1.8	2.2	2	-	-
Gzmb	granzyme B	14939	3.7	4.7	4.1	4.2	2.4	-
Gadd45b	growth arrest and DNA-damage-inducible 45 beta	17873	1.3	1.5	1.2	1.5	-	-
Gbp1	guanylate binding protein 1	14468	2.1	2.7	1.6	2.5	-	-
Gbp11	guanylate binding protein 11	634650	3.9	4.5	2.9	4.2	1.6	-
Gbp2	guanylate binding protein 2	14469	2.3	2.8	1.5	2.7	-	-
Gbp3	guanylate binding protein 3	55932	1.6	1.8	1.2	1.6	-	-
Gbp4	guanylate binding protein 4	17472	2.1	2.7	1.3	2.4	-	-
Gbp5	guanylate binding protein 5	229898	2	2.8	1.3	2.2	1.1	-
Gbp7	guanylate binding protein 7	229900	1.6	2.2	1.2	1.7	-	-
Gbp10	guanylate-binding protein 10	626578	2.1	3.2	1.3	1.9	-	-
Gbp8	guanylate-binding protein 8	76074	1.3	1.4	1.4	2.1	-	-
Gbp9	guanylate-binding protein 9	236573	1.2	1.9	1	1.3	-	-
Hp	haptoglobin	15439	1.2	1.8	1.7	1.9	1.3	1.3
Hspa1b	heat shock protein 1B	15511	2.3	2.9	2.4	2.5	-	-
H2-Q4	histocompatibility 2, Q region locus 4	15015	1	1.2	-	1.2	-	-
H2-Q6	histocompatibility 2, Q region locus 6	110557	1	1	-	1.1	-	-
H2-T22	histocompatibility 2, T region locus 22	15039	1	1.3	-	-	-	-
H2-T23	histocompatibility 2, T region locus 23	15040	1	1.3	-	1	-	-
Irgm1	immunity-related GTPase family M member 1	15944	1.4	2.3	1.1	1.4	-	-
Irgm2	immunity-related GTPase family M member 2	54396	1	1.7	-	1.2	-	-
Irg1	immunoresponsive gene 1	16365	2.6	2.5	1.9	2.5	1.6	-
Ifi202b	interferon activated gene 202B	26388	2.4	2.6	2.1	2.2	-	-
Ifi204	interferon activated gene 204	15951	3.3	4	3.2	3.9	-	-
Igtp	interferon gamma induced GTPase	16145	1.4	2.2	1	1.8	-	-
Ifitm6	interferon induced transmembrane protein 6	213002	1.8	1.9	2.4	2.3	1.6	1.5
Ifih1 (MDA5)	interferon induced with helicase C domain 1	71586	1.3	2.1	1.2	1.2	-	-
Iigp1	interferon inducible GTPase 1	60440	1.7	2.9	1.2	1.9	-	-
Irf1	interferon regulatory factor 1	16362	-	1.3	-	1.1	-	-
Irf7	interferon regulatory factor 7	54123	1.7	2.9	1.7	1.1	-	-
Ifi35	interferon-induced protein 35	70110	1.1	1.5	1.1	1.2	-	-
Ifi44	interferon-induced protein 44	99899	2	2.5	1.8	1.6	-	-
Ifi44l	interferon-induced protein 44 like	15061	2.1	2.9	2	2	-	-
Ifit1	interferon-induced protein with tetratricopeptide repeats 1	15957	2.2	3.3	2	1.7	-	-
Ifit2	interferon-induced protein with tetratricopeptide repeats 2	15958	1.8	2.5	1.8	1.7	-	-
Il1a	interleukin 1 alpha	16175	1.9	2	2.2	2.3	1.8	-
Il1f9	interleukin 1 family, member 9	215257	1.3	1.6	1.7	1.8	1.2	1
Il1rn	interleukin 1 receptor antagonist	16181	1.3	1.1	-	1.2	-	-
Il12rb1	interleukin 12 receptor, beta 1	16161	2	2.4	1.7	2.1	1.1	-
Il12rb2	interleukin 12 receptor, beta 2	16162	1.2	1.3	1.3	1.3	-	-
Il15	interleukin 15	16168	1.1	1.3	1.2	1	-	-
Il15ra	interleukin 15 receptor, alpha chain	16169	1.8	1.9	1.5	1.8	1.2	-
Il18bp	interleukin 18 binding protein	16068	1.2	1.7	1.3	1.5	-	-
Il2ra	interleukin 2 receptor, alpha chain	16184	1.1	1.2	-	1.3	1.1	-
Il33	interleukin 33	77125	-	1	-	1.1	-	-
Isg15	ISG15 ubiquitin-like modifier	100038882	1.6	2.2	1.5	1.4	-	-
Klrk1	killer cell lectin-like receptor subfamily K, member 1	27007	1.5	1.7	1.6	1.5	-	-
Lgals9	lectin, galactose binding, soluble 9	16859	1.2	1.6	1.1	1.2	-	-
Lgals3bp	lectin, galactoside-binding, soluble, 3 binding protein	19039	1.2	1.7	1.1	-	-	-
Lilrb4	leukocyte immunoglobulin-like receptor, subfamily B, member 4	14728	1.6	1.5	1.7	1.7	1.1	-
Lcn2	lipocalin 2	16819	1.4	1.6	2.2	2.2	1.4	1.7
Ly6a	lymphocyte antigen 6 complex, locus A	110454	1.7	1.9	1.2	1.3	-	-
Ly6c1	lymphocyte antigen 6 complex, locus C1	17067	1.8	2.2	1.9	1.9	-	-
Ly6c2	lymphocyte antigen 6 complex, locus C2	100041546	1.3	1.6	1.3	1.1	-	-
Ly6g	lymphocyte antigen 6 complex, locus G	546644	-	1.7	2.5	2.2	-	-
Msr1	macrophage scavenger receptor 1	20288	2.3	2	2	2.2	1.4	1.1
Mmp13	matrix metallopeptidase 13	17386	2.7	2.6	2.4	2.4	1.5	-
Mmp19	matrix metallopeptidase 19	58223	1.9	2	2	2	1.5	1.2
Mmp25	matrix metallopeptidase 25	240047	-	1	-	1.1	-	-
Mmp8	matrix metallopeptidase 8	17394	2.7	3	3.1	3.4	2.5	2.8
Ms4a4a	membrane-spanning 4-domains, subfamily A, member 4A	666907	2.6	2.7	2.6	2.5	1.5	1.1
Ms4a4c	membrane-spanning 4-domains, subfamily A, member 4C	64380	1.2	1.5	1.2	1	-	-
Ms4a4d	membrane-spanning 4-domains, subfamily A, member 4D	66607	1.6	1.5	1.6	1.8	-	-
Ms4a6c	membrane-spanning 4-domains, subfamily A, member 6C	73656	1.1	1.1	1.2	1.2	-	-
Ms4a6d	membrane-spanning 4-domains, subfamily A, member 6D	68774	3.2	3.5	3.1	3.3	-	-
Ms4a7	membrane-spanning 4-domains, subfamily A, member 7	109225	1.7	1.4	2	1.9	1.4	-
Mlkl	mixed lineage kinase domain-like	74568	2.1	2.7	2	2.3	-	-
Mnda	myeloid cell nuclear differentiation antigen	381308	1.8	1.8	1.6	1.7	-	-
Mndal	myeloid nuclear differentiation antigen like	1E + 08	1.1	1.3	1.1	1.2	-	-
Mx1	myxovirus (influenza virus) resistance 1	17857	3.2	3.9	3	2.8	-	-
Mx2	myxovirus (influenza virus) resistance 2	17858	2.7	3.7	2.1	2.2	-	-
Nampt	nicotinamide phosphoribosyltransferase	59027	1.7	1.9	1.5	2	-	-
Nmi	N-myc (and STAT) interactor	64685	1.3	1.6	1.1	1.5	-	-
Prf1	perforin 1 (pore forming protein)	18646	1.2	1.4	1.1	1.2	-	-
Phf11a	PHD finger protein 11A	219131	1.2	1.4	1.1	1.1	-	-
Phf11b	PHD finger protein 11B	236451	2	1.8	1.7	1.8	-	-
Phf11c	PHD finger protein 11C	628705	2	2.4	1.7	1.7	-	-
Phf11d	PHD finger protein 11D	219132	2.4	2.9	2.4	2.4	1.1	-
Plac8	placenta-specific 8	231507	1.3	1.7	-	1.3	-	-
Parp10	poly (ADP-ribose) polymerase family, member 10	671535	1.2	1.5	-	1.2	-	-
Parp12	poly (ADP-ribose) polymerase family, member 12	243771	1.5	2.5	1.3	1.6	-	-
Parp14	poly (ADP-ribose) polymerase family, member 14	547253	1.1	1.7	-	1	-	-
Parp9	poly (ADP-ribose) polymerase family, member 9	80285	1.3	1.9	1.1	1.2	-	-
Psme1	proteasome (prosome, macropain) 28 subunit, alpha	19186	-	1.1	-	1	-	-
Psma7	proteasome (prosome, macropain) subunit, alpha type 7	26444	-	1.1	-	1.1	-	-
Psmb10	proteasome (prosome, macropain) subunit, beta type 10	19171	1.1	1.4	-	1.3	-	-
Psmb8	proteasome (prosome, macropain) subunit, beta type 8 (large multifunctional peptidase 7)	16913	-	1.4	-	1.1	-	-
Pyhin1	pyrin and HIN domain family, member 1	236312	1.7	1.8	1.5	1.4	-	-
Pydc3	pyrin domain containing 3	100033459	1.9	2.4	1.7	1.5	-	-
Pydc4	pyrin domain containing 4	623121	2.9	3.4	2.2	1.9	-	-
Ppa1	pyrophosphatase (inorganic) 1	67895	1.3	2.1	-	1.5	-	-
Pdk4	pyruvate dehydrogenase kinase, isoenzyme 4	27273	1.5	1.1	1.7	1.4	1.8	1.8
Rtp4	receptor transporter protein 4	67775	1.4	2.2	1.2	1.1	-	-
Retnlg	resistin like gamma	245195	1.1	1.3	1.5	1.6	1.4	1.6
Rnf19b	ring finger protein 19B	75234	-	1.2	-	1.1	-	-
Rnf213	ring finger protein 213	672511	1.4	2.2	1	1	-	-
Slfn1	schlafen 1	20555	1.8	1.9	1.3	1.5	-	-
Slfn3	schlafen 3	20557	1.3	1.7	1.5	1.7	-	-
Slfn4	schlafen 4	20558	1.9	3	2	1.8	-	-
Slfn5	schlafen 5	327978	1.5	2.4	1.4	1.1	-	-
Slfn9	schlafen 9	237886	1.5	2.4	1.4	1.7	-	-
Serpina3f	serine (or cysteine) peptidase inhibitor, clade A, member 3 F	238393	1.9	2.7	1.3	2.2	1.3	-
Serpinb9	serine (or cysteine) peptidase inhibitor, clade B, member 9	20723	1.3	1.1	1.1	1.4	-	-
Serpinb9b	serine (or cysteine) peptidase inhibitor, clade B, member 9b	20706	1.2	1	1.1	-	-	-
Serpine1	serine (or cysteine) peptidase inhibitor, clade E, member 1	18787	1.1	-	-	1	-	-
Stat2	signal transducer and activator of transcription 2	20847	1.4	1.9	1.2	1.5	-	-
Slamf8	SLAM family member 8	74748	-	1.1	-	1.2	-	-
Slc15a3	solute carrier family 15, member 3	65221	-	1.3	-	1.1	-	-
Slc25a22	solute carrier family 25 (mitochondrial carrier, glutamate), member 22	68267	-	1.2	1	1.2	-	-
Socs1	suppressor of cytokine signaling 1	12703	1.6	2.2	-	1.9	-	-
Socs2	suppressor of cytokine signaling 2	216233	1	1.4	-	1.8	-	-
Tgtp1	T cell specific GTPase 1	21822	-	1.4	-	1.1	-	-
Tgtp2	T cell specific GTPase 2	1.00E + 08	1.6	2.7	-	1.5	-	-
Trex1	three prime repair exonuclease 1	22040	-	1.2	-	1	-	-
Timp1	tissue inhibitor of metalloproteinase 1	21857	2.8	2.9	2.4	2.9	1.8	1.6
Tlr13	toll-like receptor 13	279572	1.4	1	1.7	1.5	1	-
Tlr3	toll-like receptor 3	142980	1	1.4	1.2	1.1	-	-
Tlr7	toll-like receptor 7	170743	1.1	1.2	1.2	-	-	-
Tlr8	toll-like receptor 8	170744	1.1	1.1	1.3	1	-	-
Trafd1	TRAF type zinc finger domain containing 1	231712	1.1	1.6	1	1.1	-	-
Trem3	triggering receptor expressed on myeloid cells 3	58218	1.1	1.4	1.2	1.5	-	-
Trim12c	tripartite motif-containing 12C	319236	-	1.5	1.2	1.3	-	-
Trim21	tripartite motif-containing 21	20821	1.1	1.3	-	1.4	-	-
Trim30a	tripartite motif-containing 30A	20128	1.3	2.1	-	1.1	-	-
Trim30c	tripartite motif-containing 30C	434219	2.6	3.4	2.5	2.2	-	-
Trim30d	tripartite motif-containing 30D	209387	3.1	3.5	3.4	2.7	1.4	-
Wars	tryptophanyl-tRNA synthetase	22375	1.1	1.6	-	1.4	-	-
Tnfsf10	tumor necrosis factor (ligand) superfamily, member 10	22035	2	2.3	2	1.9	-	-
Usp18	ubiquitin specific peptidase 18	24110	2.2	3.1	2	1.8	-	-
Zbp1	Z-DNA binding protein 1	58203	1.9	2.7	1.3	1.6	-	-

*Italics*: Genes induced by LSDV alone

*Italics and underlined*: Genes induced by CNPV, FWPV and MVA only, and not LSDV

**Bold**: Genes induced by Avipoxviruses only (CNPV, FWPV, FeP2 and/ or PEPV)

***Bold and Italics***: Genes induced by FWPV only

**Bold and underlined**: Genes induced by CNPV only

*Underlined*: Genes induced by LSDV and MVA, but not by the Avipoxviruses

Twenty six genes involved in the host immune/defence response were up-regulated only in avipoxvirus-infected mouse spleens (Table 2). The only avipoxvirus-specific gene that was up-regulated by all four avipoxviruses was the macrophage receptor with collagenous structure (Marco) gene which has been shown to suppress early inflammatory responses to virus infection [37]. There were, however, 9 additional genes which were up-regulated by both CNPV and FWPV that were not induced by the other viruses. These included the chemokine Ccl6 which promotes immune cell activation and recruitment [38] and the immunoglobulin heavy chain genes, Ighg (IgG) and Ighg3 (IgG3) (Table 2). Amongst these 26 avipoxvirus-specific genes, 14 were exclusively up-regulated by FWPV. The Nod-like receptor, NLR, Nod1, which has been shown to be augmented in response to virus-induced production of type I IFNs [39] was exclusively up-regulated by FWPV. Four genes were uniquely up-regulated in CNPV-infected mice including the immunoglobulin heavy chain gene, Ighm (IgM), lymphocyte antigen 96 (Ly96), proteasome maturation protein (Pomp) and Cathepsin L (Ctsl) (Table 2). PePV and FeP2 induced very little immune activation according to this microarray analysis.

Four genes were up-regulated by LSDV and MVA that were not induced by the avipoxviruses in mice (Table 2), namely the Moloney Leukemia Virus 10 (Mov10) gene, hematopoietic SH2 domain containing protein (Hsh2d), poly (ADP-ribose) polymerase family, member 11 (Parp11) and schlafen 8 (Slfn8). No genes were uniquely up-regulated in response to MVA infection (Table 2).

#### Down-regulated immune related genes

Selected down-regulated genes involved in the immune response are listed in Table 3. Full lists of down-regulated genes in response to each virus are given in Additional file 1. MVA and LSDV induced the down regulation of several genes that were not affected in avipoxvirus-infected spleens. These included three forms of the chemokine CCL21 (Ccl21a, Ccl21b, Ccl21c) which are potent chemoattractants for lymphocytes and dendritic cells [40] (Table 3). Furthermore, MVA and LSDV down regulate the high affinity IgM and IgA F_C_ receptor Fcamr. Fcamr is the receptor for the F_C_ fragment of immunoglobulins IgA and IgM [41]. Interestingly, MVA, LSDV, FWPV and CNPV all down regulate the gene encoding the murine homolog for DC-specific ICAM-3–grabbing nonintegrin (DC SIGN) (Cd209a), and MVA and LSDV down regulate an additional DC SIGN homolog, CD209b (SIGNR1) (Table 3). LSDV uniquely down regulates CD59a, which is the primary regulator of complement membrane attack in mouse [42] and CD7 which is expressed on T- and NK cells, and on cells in the early stages of T, B, and myeloid cell differentiation [43]. LSDV also uniquely down regulates the immunoglobulin kappa chain complex (IgK) amongst other immune related genes (Table 3). TLR11 is down-regulated by MVA alone (Table 3).

**Table 3:** Selection of down-regulated genes in mouse spleens in response to MVA, LSDV, CNPV, FWPV, PEPV and FeP2. Differences in Log_2_ Fold Changes (between each virus and the control) are depicted

Symbol	Name	Entrez	MVA	LSDV	CNPV	FWPV	PEPV	FeP2
**Genes down-regulated by LSDV ALONE**								
***Adamdec1***	*ADAM-like, decysin 1*	*58860*	*-*	*−1.1*	*-*	*-*	*-*	*-*
***Cd59a***	*CD59a antigen*	*12509*	*-*	*−1.2*	*-*	*-*	*-*	*-*
***Cd7***	*CD7 antigen*	*12516*	*-*	*−1.1*	*-*	*-*	*-*	*-*
***Esm1***	*endothelial cell-specific molecule 1*	*71690*	*-*	*−1.1*	*-*	*-*	*-*	*-*
***Igfbp3***	*insulin-like growth factor binding protein 3*	*16009*	*-*	*−1*	*-*	*-*	*-*	*-*
***Igk***	*immunoglobulin kappa chain complex*	*243469*	*-*	*−1*	*-*	*-*	*-*	*-*
***Lilra5***	*leukocyte immunoglobulin-like receptor, subfamily A (with TM domain), member 5*	*232801*	*-*	*−1.1*	*-*	*-*	*-*	*-*
***Prkcg***	*protein kinase C, gamma*	*18752*	*-*	*−1*	*-*	*-*	*-*	*-*
**Genes down-regulated by MVA alone**								
**Ctsf**	**cathepsin F**	**56464**	**−1.1**	**-**	**-**	**-**	**-**	**-**
**Depdc1a**	**DEP domain containing 1a**	**76131**	**−1.1**	**-**	**-**	**-**	**-**	**-**
**Diap3**	**diaphanous homolog 3 (Drosophila)**	**56419**	**−1.1**	**-**	**-**	**-**	**-**	**-**
**Hmmr (CD168)**	**hyaluronan mediated motility receptor (RHAMM)**	**15366**	**−1**	**-**	**-**	**-**	**-**	**-**
**Tlr11**	**toll-like receptor 11**	**239081**	**−1**	**-**	**-**	**-**	**-**	**-**
**Genes down-regulated by lsdv and MVA, BUT NOT BY CNPV and FWPV**								
**Ccl21a**	chemokine (C-C motif) ligand 21A (serine)	18829	−1.3	−1.7	-	-	-	-
**Ccl21b**	chemokine (C-C motif) ligand 21B (leucine)	100042493	−1.2	−1.7	-	-	-	-
**Ccl21c**	chemokine (C-C motif) ligand 21C (leucine)	65956	−1.2	−1.6	-	-	-	-
**Kel**	Kell blood group	23925	−1.5	−1.5	-	-	-	-
**Slc12a2**	solute carrier family 12, member 2	20496	−1	−1	-	-	-	-
**Timd4**	T cell immunoglobulin and mucin domain containing 4	276891	−1.1	−1.3	-	-	-	-
**Genes down-regulated by CNPV, FWPV AND MVA ONLY, and not LSDV.**								
***Tspan33***	*tetraspanin 33*	*232670*	*−1.6*	*-*	*−1.3*	*−1.1*	*-*	*-*
**OTHER**								
**Abca9**	ATP-binding cassette, sub-family A (ABC1), member 9	217262	−1.3	−1.5	-	−1.1	-	-
**Aplnr**	apelin receptor	23796	−1.4	−1.5	−1.2	−1.2	-	-
**Cd209a**	CD209a antigen	170786	−1.7	−2.2	−1.1	−1.5	-	-
**Cd209b**	CD209b antigen	69165	−1.2	−1.3	-	-	-	-
**Cldn13**	claudin 13	57255	−1.5	−1.2	−1.1	-	-	-
**Emr4**	EGF-like module containing, mucin-like, hormone receptor-like sequence 4	52614	−1.4	−1.8	−1.1	−1.5	−1.3	-
**Fcamr**	Fc receptor, IgA, IgM, high affinity	64435	−1.1	-	−1.5	-	-	-
**Fcer2a**	Fc receptor, IgE, low affinity II, alpha polypeptide	14128	−2.6	−3	−2.4	−2.5	−2.2	−1.3
**H2-M2**	histocompatibility 2, M region locus 2	14990	−1	−1.4	−1	−1	−1.1	-
**Hs3st2**	heparan sulfate (glucosamine) 3-O-sulfotransferase 2	195646	−1.4	−1.3	−1	−1	-	-
**Ifi27l1**	interferon, alpha-inducible protein 27 like 1	52668	−1.4	−1.3	-	-	-	-
**Mgst3**	microsomal glutathione S-transferase 3	66447	−1.6	−1	-	−1	-	-
**Slc16a10**	solute carrier family 16 (monocarboxylic acid transporters), member 10	72472	−1.4	-	-	−1.1	-	-
**Slc2a4**	solute carrier family 2 (facilitated glucose transporter), member 4	20528	−1.7	−1	-	−1.1	-	-
**Slc38a5**	solute carrier family 38, member 5	209837	−1.4	−1.2	−1	-	-	-
**Slc6a20a**	solute carrier family 6 (neurotransmitter transporter), member 20A	102680	−1.4	−1.3	−1	−1	-	-
**Tfrc**	transferrin receptor	22042	−1.2	-	-	-	-	-
**Tspan8**	tetraspanin 8	216350	−1.4	-	-	−1	-	-

*Italics* genes down-regulated by LSDV alone

*Bold* Genes down-regulated by MVA alone

*Underlined* genes down-regulated by LSDV and MVA, but not by avipoxviruses

*Italics and underlined* genes down-regulated by CNPV, FWPV and MVA only, and not LSDV

#### Type I interferon response

**Fig. 3:**
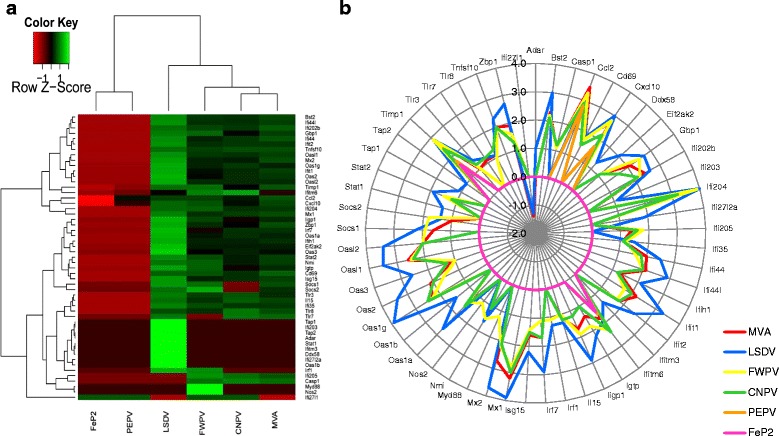
Heatmap (**a**) and radial plot (**b**) depicting the differences in the type I Interferon response induced by canarypox virus (CNPV), pigeonpox (FeP2), fowlpox virus (FWPV), lumpy skin disease virus (LSDV), modified vaccinia Ankara (MVA) and penguinpox virus (PEPV). Fig. 3. **a** represents the log_2_ fold change (FC) induction of the different genes up-regulated in the six samples compared to the control. A log_2_FC of 0 is given where genes are not present over the cut off (±1). Unsupervised hierarchical clustering of the samples is represented by dendograms. Clustering analysis and heatmap was performed in the R package, gplots (Warnes, 2009). Fig. 3. **b** shows a radial plot depicting the magnitude and breadth of the type I interferon response induced by the six viruses. The distance from the centre of the plot indicates log2-fold change (ranging from −2 to 4)

While many of the immunity related genes listed in Table 2 are regulated in some way by Type I Interferons, in order to characterise and compare the differences in the Type I IFN-regulated responses between MVA, LSDV, FWPV, CNPV, FeP2 and PEPV at 24 h, we analyzed a selection of genes known to be involved in the IFN response [44–46] (Fig. 3). This figure clearly shows that LSDV induces the greatest IFN response compared to the other viruses (Fig. 3).

#### Caspases

**Fig. 4:**
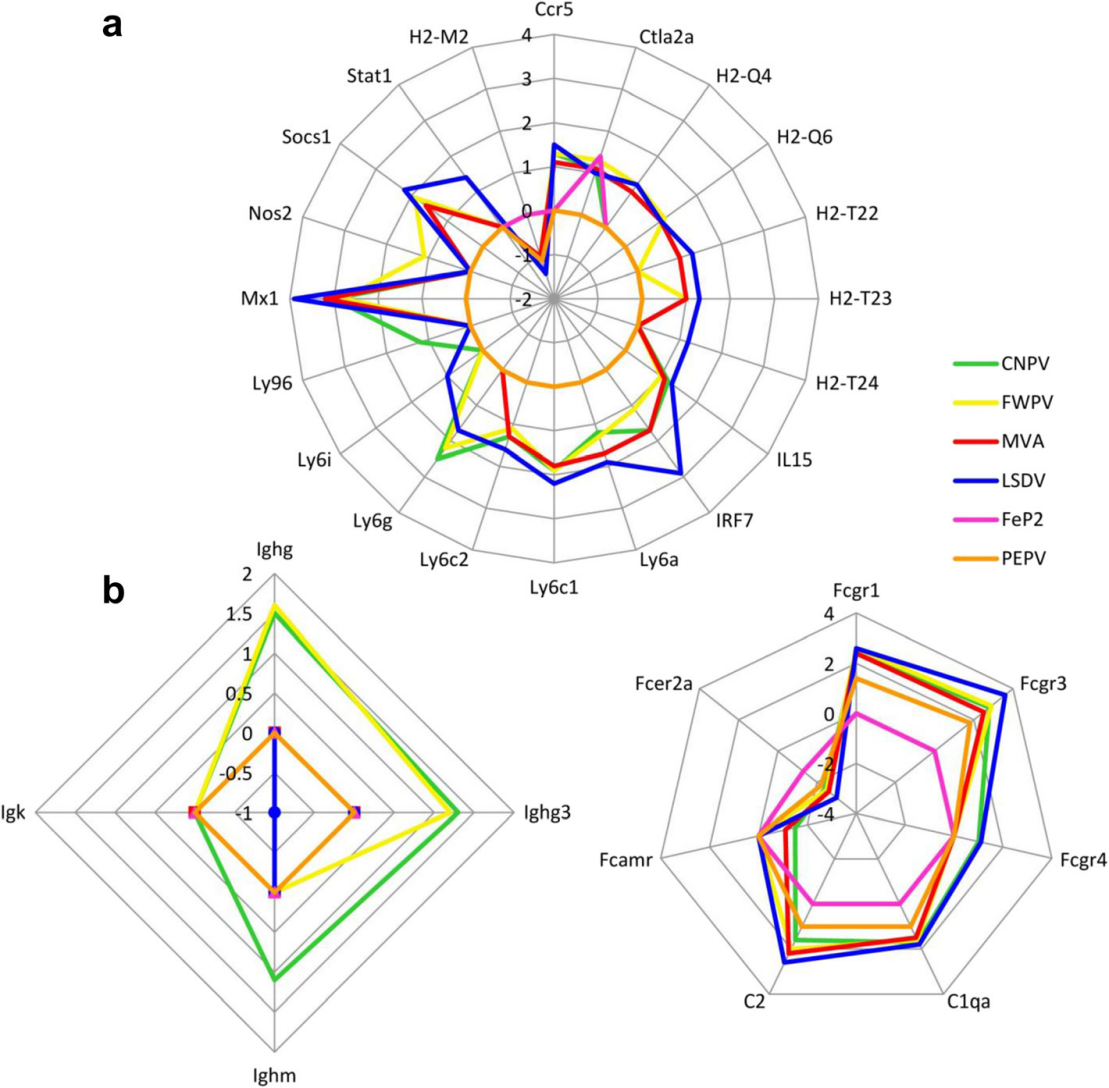
**a** T-cell specific responses and 4**b**) B-cell specific responses up- or down-regulated in mouse spleens in response to canarypox virus (CNPV), pigeonpox (FeP2), fowlpox virus (FWPV), lumpy skin disease virus (LSDV), modified vaccinia Ankara (MVA) and penguinpox virus (PEPV). The log_2_ fold changes of significantly differentially expressed (*p* value ≤ 0.05) genes involved in the respective types of responses are compared. A value of 0 indicates that no change was observed compared to mock infected mouse spleens. A positive value depicts upregulated genes and a negative value depicts down-regulated genes

MVA, FWPV and CNPV all up-regulated the protease caspase 1 (casp1) whereas LSDV did not (Fig. 4). MVA, FWPV, CNPV and LSDV significantly up-regulated caspase 4 (casp 4) (historically called caspase 11 in the mouse) (Table 2). The SA avipoxviruses, FeP2 and PEPV did not affect gene regulation of any caspase genes.

#### B- and T-cell specific responses

The up- and down-regulated genes involved in B cell and T cell responses induced by the viruses in this study were compared (Fig. 4). Fig. 4a highlights the regulated genes that are involved in the T cell response. FeP2 and PEPV regulated only one gene each involved in this response, Ctla2a and major histocompatibility (MHC) class I gene, H2-M2, respectively. The other four viruses differentially regulated several MHC class I genes amongst others (Fig. 4a). As highlighted in Table 2, the avipoxviruses, CNPV and FWPV, exclusively up regulate immunoglobulin heavy chain genes, (Ighg (IgG) and Ighg3 (IgG3)) with CNPV inducing a third, Ighm (IgM) (Fig. 4b). LSDV down regulates the immunoglobulin kappa (IgK) chain complex (Fig. 4b). In addition to these, the poxviruses differentially regulate genes for F_C_ receptors and complement (Fig. 4b).

#### Comparison of early poxvirus-induced immune responses to innate molecular signatures of published candidate vaccine vectors

We compared the gene expression induced by each of the 6 poxviruses at 24 h to selected correlates of protection and molecular signatures from previously published studies (Table 4). The induction of multiple PRRs has been shown to activate different immune pathways and thereby induce a more polyvalent immune response [47, 48]. We identified differential expression of several genes involved in pathogen recognition (Table 4). Several genes are common to the innate and adaptive immune responses induced by the poxviruses analysed here and other viral vectors analysed elsewhere (Table 4).

**Table 4:** Comparison of early poxvirus-induced immune responses to selected innate molecular signatures of existing vaccine vectors. Differences in Log_2_ Fold Changes (between each virus and the control) are depicted

	description	MVA	LSDV	CNPV	FWPV	PEPV	FeP2	Evidence	References
**Innate immune response**									
***Pathogen recognition***									
**Tlr13**	toll-like receptor 13	1.4	1.0	1.7	1.5	1.0	-		
**Tlr3**	toll-like receptor 3	1	1.4	1.2	1.1	-	-	Merck Ad5/HIV	[22]
**Tlr7**	toll-like receptor 7	1.1	1.2	1.2	-	-	-	YF-17D, LAIV	[20, 21]
**Tlr8**	toll-like receptor 8	1.1	1.1	1.3	1.0	-	-	Merck Ad5/HIV	[22]
**Tlr11**	toll-like receptor 11	−1.0	-	-	-	-	-		
**Ddx58 (RIG-I)**	RIG-I-like receptor	-	1.3	-	-	-	-	YF-17D	[20]
**Cd209a (DC SIGN)**	CD209a antigen	−1.7	−2.2	−1.1	−1.5	-	-		
**Cd209b (DC SIGN)**	CD209b antigen	−1.2	−1.3	-	-	-	-		
**Ifih1 (MDA5)**	RIG-I-like receptor	1.3	2.1	1.2	1.2	-	-	YF-17D	[20]
**Zbp1 (DAI)**	cytoplasmic double-stranded DNA sensor	1.9	2.7	1.3	1.6	-	-		
**Dhx58 (LGP2)**	RIG-I-like receptor	1.7	2.5	1.6	1.5	-	-	YF-17D	[20]
**Eif2ak2 (PKR)**	eukaryotic translation initiation factor 2-alpha kinase 2 (protein kinase R)	1.1	2.1	1.1	1.1	-	-	YF-17D	[20]
***Genes associated with the innate immune response of viral vectors***									
**Cxcl10 (IP-10)**	chemokine (C-X-C motif) ligand 10	2.7	3	2.2	2.8	1.7	-	Significantly upregulated in response to YF-17D, Merck Ad5/HIV, TIV	[20−22]
**Mx1**	myxovirus (influenza virus) resistance 1	3.2	3.9	3	2.8	-	-	YF-17D	[20]
**Il-1α**	interleukin 1 alpha	1.9	2	2.2	2.3	1.8	-	Significantly upregulated in response to YF-17D	
**Isg15**	ISG15 ubiquitin-like modifier	1.6	2.2	1.5	1.4	-	-	Merck Ad5/HIV	[22]
**Stat1**	signal transducer and activator of transcription 1	-	1.4	-	-	-	-	YF-17D, Merck Ad5/HIV, LAIV	[20−22]
**Cxcl11 (I-TAC)**	chemokine (C-X-C motif) ligand 11	4.5	4.4	3.4	4.3	1.5	-	Merck Ad5/HIV	[22]
**Ccr5**	chemokine (C-C motif) receptor 5	1.1	1.5	1.3	1.3	-	-	Merck Ad5/HIV	[22]
**Gbp7**	guanylate binding protein 7	1.6	2.2	1.2	1.7	-	-	Merck Ad5/HIV	[22]
**Irf1**	interferon regulatory factor 1	-	1.3	-	1.1	-	-	Merck Ad5/HIV	[22]
**Stat2**	signal transducer and activator of transcription 2	1.4	1.9	1.2	1.5	-	-	LAIV	[21]
**Irf7**	interferon regulatory factor 7	1.7	2.9	1.7	1.1	-	-	LAIV	[21]
**Casp1**	caspase 1	1.1	-	1.2	1.1	-	-		
**Adaptive immune response**									
**B cell related responses**									
**Ighg**	Immunoglobulin heavy chain (gamma polypeptide)	-	-	1.5	1.6	-	-		
**Ighg3**	Immunoglobulin heavy constant gamma 3	-	-	1.3	1.2	-	-	TIV, correlated with decreased risk of HIV-1 infection in the RV144 trial ALVAC-HIV(vCP1521)	
**Ighm**	immunoglobulin heavy constant mu	-	-	1.1	-	-	-	positively correllates with antibody response to TIV	[21]
**Igk**	immunoglobulin kappa chain complex	-	−1.0	-	-	-	-	positively correllates with antibody response to TIV	[21]
**T cell related responses**									
**Gzmb**	granzyme B	3.7	4.7	4.1	4.2	2.4	-	expressed by CD8+ T cells in response to YF-17D	[20]
**Ccr5**	chemokine (C-C motif) receptor 5	1.1	1.5	1.3	1.3	-	-	expressed by CD8+ T cells in esponse to YF-17D	[20]
**Ccl2 (MCP1)**	chemokine (C-C motif) ligand 2	3.5	3.3	2.9	3.3	2.8	-	predicted the magnitude of the CD8+ T cell response to Merck Ad5/HIV	[22]

*HIV* human immunodeficiency virus, *LAIV* live attenuated influenza vaccine, *TIV* trivalent influenza vaccine, *YF-17D* Yellow fever vaccine, *Merck Ad5/HIV* Merck’s Adenovirus subtype 5-based HIV vaccine

## Discussion

Novel avipoxviruses have been isolated in South Africa with the goal of identifying novel vaccine vectors [26, 27]. It is desirable to be able to select potential avipoxvirus vaccine vectors without going through the process of making recombinant viruses and testing immunogenicity in animal models. In this study, we compared the gene expression profiles in mouse spleens 24 h after infection with six poxviruses from 3 different genera. All the viruses were grown in eggs and the same purification methodology used. There have been no comparisons of host responses to these six different poxviruses. One of the aims of this study was to determine if one could select novel poxvirus vaccine vectors based on the transcriptome analysis. It was hypothesized that if the transcriptomes were identical then it was unlikely that they would differ as vaccine vectors. The complex model of the mouse spleen was selected because the spleen is rich in immune cells and the immune response is a complex interaction between different types of cells and their proteins which would not be reflected in in vitro models. Although in vitro expression studies have provided useful information, gene expression profiles performed in cell culture may not accurately reflect the changes that occur as result of infection in vivo. It is anticipated that different poxviruses will have different properties that will relate to their ability to act as adjuvants driving the immune response to the vaccine protein towards a particular type of immune response.

Unsupervised hierarchical clustering differentiates between the observed responses to the six poxviruses, grouping CNPV and MVA together and FWPV in a separate cluster (more closely related to CNPV and MVA than to LSDV) (Fig. 1). FeP2 and PEPV group together in a cluster that is separate from the other four viruses (Fig. 1). This grouping is quite different from phylogenetic relationships established by DNA sequence comparisons [27]. It is also not dependent on the viral morphogenesis in non-permissive cells. PEPV and FWPV have been demonstrated to infect mammalian cells [28] and progress to a late stage in morphogenesis [28, 49] whereas FeP2 [50] and CNPV have a block prior to DNA replication [51]. It is noted that infectivity studies have not been done in mouse spleens and that this may be different to published data on other mammalian cells. A further study is needed to determine if PEPV and FeP2 infect the same number of cells in the mouse spleen as the other viruses.

Amongst the four avipoxviruses analysed here, the greatest difference in host responses was expected between CNPV and FWPV, as on a genomic level, these viruses are significantly divergent with amino acid identity between ORF homologues (55–74 %) being similar to that observed between different ChPV genera [52]. We did not expect to see such significant differences between the host responses induced by FeP2 and PEPV which share 94.4 % nucleotide identity with each other and 85.3 and 84.0 % nucleotide identity with FWPV respectively [27]. Since avipoxviruses are restricted to avian hosts, one would anticipate fewer differences between the mammalian host responses induced by them as it is highly likely that their proteins are not as functional in mammalian cells as those of MVA and LSDV. However we show that three relatively closely related avipoxviruses (FWPV, FeP2 and PEPV) induce significant differences in gene expression in the host. FWPV induced the strongest host response in mice whereas FeP2 infection resulted in remarkably little change in host gene expression. A vaccine vector with low host reactivity, such as observed with PEPV or FeP2, might have the advantage of being a good vector to include adjuvant genes to enhance immunogenicity. Further investigation is needed to determine if there would be a good immune response to foreign antigens expressed by these vectors.

Previous in vitro studies with VACV, MVA and NYVAC have shown more host genes to be down-regulated than up-regulated [11–14]; however, this was not the case in other poxviruses, including cowpox virus (CPXV) [53], monkeypox virus (MPXV) [53] or ALVAC [15]. Unlike our study, done in a mouse model, all these studies were done in cell culture. We show that fewer genes are down-regulated than up-regulated in response to in vivo infection at 24 h post infection (Table 1). For each poxvirus tested several of the dysregulated mouse genes are not yet annotated suggesting biological roles for unannotated genes and highlighting the importance of further functional analysis and annotation of the mouse genome. Contrary to in vitro studies, MVA caused more transcripts to be up-regulated than down-regulated in mouse spleens. MVA infection did, however, result in a greater number of down-regulated transcripts compared to LSDV, FWPV and CNPV.

MVA and LSDV induced the down-regulation of several genes that were not affected by the avipoxviruses tested including three forms of the chemokine CCL21 (Ccl21a, Ccl21b, Ccl21c) which are potent chemoattractants for lymphocytes and dendritic cells [40] (Table 3). VACV A41L encodes a chemokine binding protein which binds and inhibits CCL21 [54] and deletion mutants lacking the A41L gene, induce stronger virus-specific CD8+ T-cell responses [54, 55]. LSDV does not have a homolog of the A41L gene; there must be other mechanisms that mammalian poxviruses have evolved to evade the effects of CCL21, which is clearly important for the host in clearing poxvirus infection. In selecting/designing a vaccine vector it would be desirable to use a virus which lacks A41L and does not down regulate CCL21 if a strong CD8+ T cell response is required.

Interestingly, MVA, LSDV, FWPV and CNPV all down-regulated the gene encoding the murine homolog for DC-specific ICAM-3–grabbing nonintegrin (DC SIGN) (Cd209a). Furthermore MVA, LSDV and FWPV down-regulated an additional DC SIGN homolog, CD209b (SIGNR1) (Table 3).

LSDV has a host-range restricted to ruminants and is currently being investigated as an HIV vaccine vector [3, 56]. LSDV caused the most significant response in mice compared to the other poxviruses, both in terms of the number of up-regulated genes and the magnitude and breadth of the type I Interferon response (Fig. 4). LSDV clustered independently from the avipoxviruses and MVA. LSDV up-regulated genes are involved in the antigen processing and presentation pathway (H2-T24, Tap1 and Tap2). Furthermore, LSDV uniquely up-regulated the gene encoding macrophage migration inhibitory factor (Mif), which is important in both macrophage function and T-cell immunity [57], and Ddx58, otherwise known as RIG-I (retinoic acid-inducible gene 1), which recognises viral RNA, activating downstream signalling pathways that facilitate type I IFN production [58]. The up-regulation of RIG-I may, in part, be responsible for the increased type I IFN response seen in LSDV-infected mice. Another one of the many genes uniquely up-regulated by LSDV was the transcription factor (Myc) that promotes growth, proliferation and apoptosis [59]. Myc has been shown to be up-regulated in response to infection with NYVAC and MVA in HeLa cells [13]. The absence of Myc up regulation in mouse spleens by MVA was unexpected. In a study done in Rhesus macaques rLSDV vector expressing an HIV polyprotein was immunogenic at a dose 1000-fold lower than that of rMVA. Both CD4+ and CD8+ responses were induced, rather than a predominance of CD4+ T cells observed typically for poxvirus vectors [3].

Both LSDV and MVA up-regulated a cellular homolog of Moloney Leukemia Virus 10 (Mov10), which has been shown to inhibit retrovirus replication and infectivity [60]. It specifically interacts with the nucleocapsid domain of HIV Gag [60], which may have implications for vaccine vectors encoding Gag proteins. Avipoxviruses may therefore be better vectors than MVA or LSDV for the expression of Gag.

CNPV and FWPV induce the up regulation of two immunoglobulin genes (Ighg and Ighg3 (IgG3)) with CNPV up regulating a third, Ighm (Fig. 4b). All six viruses down-regulated the IgE FC receptor alpha (Fcer2a) polypeptide gene. IgE is involved in allergic responses and not vaccine responses. Antibodies of the same epitope specificity but of a different subclass can have different antibody effector functions [61]. In a recent comparison of the immune responses resulting from the partially effective clinical RV144 HIV-1 trial and the ineffective VAX003 trial, it was shown that HIV-1–specific IgG3 antibodies were correlated with decreased risk of HIV-1 infection in the RV144 trial. It is suggested that the canarypox virus, ALVAC-HIV (vCP1521) prime component of RV144 may have stimulated different antibody subclasses, specifically IgG3, compared to the protein-only vaccine (VAX003) [61]. The up regulation of IgG3 specifically by FWPV and CNPV in vivo, suggests that these two avipoxvirus vectors may be involved in stimulation of the clinically important IgG3 antibody subclass. Up regulation of IgG3 was not detected in ALVAC-infected monocyte derived dendritic cells (MDDCs) [15]; this potentially significant finding is an example of the importance of in vivo testing.

Type I IFN responses have been highlighted in previous studies investigating host gene expression changes in response to different host-restricted poxviruses [14, 15]. Type I IFN induces an extensive range of interferon stimulated genes (ISGs) with various anti-viral functions (reviewed here: [62]). In concurrence with previous studies of poxvirus-induced host responses [14, 15], Type I IFN responses were initiated by MVA, LSDV, CNPV and FWPV, with LSDV inducing the strongest response in mice (Fig. 4), followed by CNPV and FWPV, with MVA inducing a relatively low IFN response. FeP2 and PEPV induced very little ISG expression. The observed enhanced type I IFN-specific and other immune responses elicited by LSDV, FWPV and CNPV compared to MVA may be due to the absence of virus-encoded immunomodulators in these viruses which could still be present in MVA. Our results suggest that LSDV may be more immunogenic than FWPV and CNPV in mice. It is not known whether this greater IFN-response induced by LSDV in comparison to avipoxviruses would lead to enhanced clearance of the virus and a decreased immune response to any potential transgenes, or whether the increased IFN response would result in an improved immune response to the transgene, should LSDV be used as a vaccine vector.

Toll-like receptors are important regulators of the innate immune system. Poxviruses are recognized by a number of different pathogen recognition receptors (PRRs) with innate immune sensing patterns differing considerably between species and even between different derivatives of the same parent species (VACV, MVA and NYVAC) [23]. In our study we show that TLR13, TLR3 and TLR8 are up-regulated by four poxviruses analysed (CNPV, FWPV, MVA and LSDV). In addition, TLR7 is up-regulated by CNPV, MVA and LSDV but not by FWPV (Table 2). Up regulation of TLR 3, which detects double stranded RNA, has been observed in response to MVA, but not NYVAC infection of MDDCs [14]. IFNs have been shown to up regulate TLR gene expression in viral infections [63]. Here we have established that CNPV, FWPV, MVA and LSDV all induce significant type I IFN responses and we suggest that the up regulation of TLR expression may be a result of this.

MVA, FWPV and CNPV all up regulate the protease Caspase 1 (Casp1) whereas LSDV does not (Fig. 4). Casp1 dependent programmed cell death (pyroptosis), unlike apoptosis, is a pro-inflammatory process that has recently been recognised as important for the control of microbial infections [64]. All of MVA, FWPV, CNPV and LSDV also significantly up-regulated caspase 4 (casp 4) (historically called caspase 11 in the mouse) (gene lists reference) which is required for the maturation of the pro-proteins of IL-1b and IL-18 (proIL-1b, proIL-18) and plays an important role in the activation of caspase-1 in inflammasome complexes, and therefore inflammation [65]. The correlation of caspase up-regulation with either apoptosis or pyroptosis is still to be assessed.

Application of the systems biology approach to vaccines and determination of innate immune signatures has proven useful in predicting the immunogenicity of the highly effective yellow fever vaccine (YF-17D) [20], the seasonal influenza vaccines [21] and the immunogenic but inefficacious Merck Ad5/HIV vaccine [22]. Several of the innate immune signatures observed in tested vaccines, were common to one or more of the poxviruses investigated here. The gene encoding monocyte chemotactic protein 1 (MCP1) (Ccl2) was up-regulated by 5 out of the 6 poxviruses (MVA, LSDV,CNPV,FWPV and PEPV). This gene was positively correlated with the CD8+ T cell response to Merck Ad5/HIV vaccination [22]. Immunoglobulin genes, Ighm (up-regulated by CNPV) and IgK (down-regulated by LSDV) were positively correlated with the antibody response to TIV influenza vaccination [21]. This suggests that the different poxviruses could be associated with different levels of antibody induction during the adaptive immune response. Based on our data we speculate that LSDV may be more suitable for a T-cell based vaccine and CNPV more suitable for the induction of an antibody response. This reflects the published data on LSDV [3] and CNPV [61].

Microarray analyses can provide important information regarding the effect of different clinically relevant viruses on host gene expression. One limitation of microarray data analysis is that as of yet there are no standardised methods of statistical analysis. It has been demonstrated previously that fold change designations and *p*-value cutoffs can significantly alter microarray interpretation [66]. Here we have chosen stringent fold change and *p*-value cutoffs (log_2_FC ±1, adjusted *p*-value < 0.05) in line with similar studies [11−14], in order to avoid false discovery and inaccurate biological inferences. We concede that in doing so, some smaller changes in gene expression may have been overlooked. Further work should entail investigating gene dysregulation at different times post infection. Also, innate immune signatures should be directly correlated with subsequent adaptive responses. Correlation of gene expression data with biological or clinical findings would be most informative.

## Conclusions

The findings presented here indicate that six, genetically diverse host-restricted poxviruses, CNPV, FWPV, FeP2, PEPV, MVA and LSDV, produce qualitatively and quantitatively distinct host responses in an in vivo mouse model. These results confirm that transcriptome analysis in a mouse model can be used to determine if poxvirus vectors differ from each other, laying the ground work for further investigation.

## Materials and methods

### Animal ethics approval

The growth of poxviruses in embyonated eggs and the mouse experiments described below were approved by the Animal Research Ethics Committee in the Faculty of Health Sciences, University of Cape Town. The approval numbers are 013/016 and 013/017 respectively.

### Viruses

MVA and wild-type CNPV were obtained from Prof. K. Dumbell’s collection at the University of Cape Town and were originally from Prof. A. Mayr (Germany). The fowlpox virus vaccine, DCEP 25 modified strain, was purchased from Merial (Country) and LSDV vaccine, Herbivac® (Ceva), was kindly donated by Deltamune (Pretoria, South Africa). FeP2 was from a Feral Pigeon (*Columba livia*) [26, 27] and PEPV from an African penguin (*Spheniscus demersus*) [27, 29]. Virus isolates were grown and titrated on the chorioallantoic membranes (CAMs) of embryonated 10–11 day old (MVA, CNPV, FWPV) or 7 day old (LSDV) Specific pathogen–free (SPF) White Leghorn chicken eggs, which were obtained from Avifarms (Pty) Ltd (Lyttelton, South Africa), using a method described previously [26]. Titrations were performed on CAMs for avipoxviruses and MVA and on Madin Darby bovine kidney (MDBK) cells for LSDV.

### Virus infection of mice

Seven week old naive female BALB/c mice were randomly divided into groups of three and each mouse was inoculated intravenously (i.v) with 10^5^ pfu/100 ul poxvirus, diluted in PBS or mock infected with PBS alone or egg extract (100 μl). The egg extract was made from uninfected CAMs, following the same extraction and purification procedure as the virus samples above. We compared the gene expression profiles of the groups of mice that were mock-infected with egg extract and PBS. No difference in gene expression was observed between the control samples. For each different virus, three groups of three mice each were inoculated. At 24 h post infection, the mice were sacrificed by cervical dislocation without anaesthesia and the spleens were harvested and placed in RNAlater (Qiagen, Venlo, Limburg, NL).

### RNA extraction

Mouse spleens were removed from RNAlater and the three spleens in each group were pooled and homogenized thoroughly using a TissueRuptor (Qiagen) in TRIzol® reagent (Life Technologies, Carlsbad, CA, USA). Total RNA was isolated using TRIzol® Plus RNA Purification Kit (Life Technologies, Carlsbad, CA, USA) with On-column PureLink® DNase treatment according to manufacturer’s instructions. RNA was resuspended in RNase free water and quality checked using the Nanodrop ND1000 (Thermoscientific, Waltham, MA, USA) and the Agilent Bioanalyzer Nano Assay (Agilent, Santa Clara, CA, USA).

### Microarray and data analysis

mRNA hybridization was performed by IMGM Laboratories GmbH (Martinsried, DE) with the Affymetrix GeneChip Mouse Gene 2.0 ST array (Affymetrix, Santa Clara, CA, USA). Data analysis was performed in R [67], using packages from the Bioconductor suite (http://www.bioconductor.org), and CRAN (http://cran.rproject.org). All R code is available in Additional file 1. Probe level data from. CEL files was normalised using the Robust Multi-array Averaging (RMA) method [68] obtained as part of the “affy” package [69] from Bioconductor, resulting in log_2_ transformed values. Boxplots, scatterplots and histogram outputs of the normalised data were obtained and checked for consistency (not shown). Data was annotated using the Mouse Gene ST 2.0 annotation data package from Bioconductor. Non-specific filtering was performed using the Genefilter package [70, 71]. This step included an intensity filter which filtered the data set such that the intensity of each gene should be > log2 (100) in at least 20 % of the samples. Secondly, a variance filter was applied such that the interquartile range of log2-intensities should be at least 0.5.

Differential gene expression was determined using a linear model approach using the R package, Limma [34]. A heatmap was made using heatmap.2 from the CRAN package gplots [72], and depicted the unsupervised hierarchical clustering based on the genes with *p*-value < 0.05 and log_2_FC above or below cutoff (±1). Venn diagrams were made using Venny [73] available at http://bioinfogp.cnb.csic.es/tools/venny/index.html. Functional analysis was performed using Database for Annotation, Visualization and Integrated Discovery (DAVID) v6.7 web-based tools (http://david.abcc.ncifcrf.gov/tools.jsp). Quantitative real time PCR was done on selected mouse genes. GAPDH, HPRT and CD51 were selected as housekeeping genes; IRF7 and Zbp1 were selected as genes which were moderately upregulated by LSDV, MVA and avipoxviruses CNPV and FWPV; and IGFbp3 was selected as a gene which was downregulated by LSDV.

## Additional file

Additional file 1:
**R code used. Supplementary file 2.** Full list of up regulated genes in mouse spleens in response to modified vaccinia Ankara (MVA), lumpy skin disease virus (LSDV), canarypox virus (CNPV), fowlpox virus (FWPV), penguinpox virus (PEPV) and pigeonpox (FeP2). **Supplementary file 3.** Full list of down regulated genes in mouse spleens in response to modified vaccinia Ankara (MVA), lumpy skin disease virus (LSDV), canarypox virus (CNPV), fowlpox virus (FWPV), pigeonpox (FeP2) and penguinpox virus (PEPV).
