# Supplementary material for: Six host-range restricted poxviruses from three genera induce distinct gene expression profiles in an in vivo mouse model
Source: BMC Genomics. 2015 Jul 8;16(1):510. doi: 10.1186/s12864-015-1659-1 (PMC4495948; doi:10.1186/s12864-015-1659-1)
Supplement: Additional file 1: — R code used. Supplementary file 2. Full list of up regulated genes in mouse spleens in response to modified vaccinia Ankara (MVA), lumpy skin disease virus (LSDV), canarypox virus (CNPV), fowlpox virus (FWPV), penguinpox virus (PEPV) and pigeonpox (FeP2). Supplementary file 3. Full list of down regulated genes in mouse spleens in response to modified vaccinia Ankara (MVA), lumpy skin disease virus (LSDV), canarypox virus (CNPV), fowlpox virus (FWPV), pigeonpox (FeP2) and penguinpox virus (PEPV). [file 12864_2015_1659_MOESM1_ESM.pdf]

Supplementary File 1

R code used

```
> #############################################################################################
> #R script for analysis of gene expression by poxviruses#
> #############################################################################################

> #set working directory
> setwd("c:/R/data/RawData")
> #view files in working directory
> dir("c:/R/data/RawData")

> #load packages
> library(affy)
> library(limma)
> library(oligo)
> library(annotate)
> library(genefilter)
> library(Biobase)
> library(RColorBrewer)
> library(multtest)
> library(ArrayTools)
> library(pathview)
> library(gage)
> library(GOstats)
> library(ReactomePA)
> library(biomaRt)
> library(GO.db)
> library(mogene20stprobeset.db)
> library(mogene20sttranscriptcluster.db)
> library(genefilter)
> library(gplots)

> #load data
> RawData <-ReadAffy

> #read in .cel files
> RawData <- read.celfiles(list.celfiles("C:/R/data/RawData", full.names = T),verbose=TRUE)

> #load phenotype data
> pd<-read.AnnotatedDataFrame("phenodata.txt",header=TRUE,row.names=1)
> pd.2<-read.table("phenodata.txt",header=T,row.names=1)
> x <- varMetadata(pd)
> x <- data.frame(x, channel = "_ALL_")
> varMetadata(pd) <- x

> #RMA normalisation (background correction, normalizing and expression calculation)
> OligoEset<-rma(RawData,target="core")
> pData(OligoEset)<-pd.2

> #boxplots
> boxplot(OligoEset, col="red",names=pd$label,las=2,main="Normalized data")

> #hist
> hist(OligoEset, fig=TRUE)

> # Scatterplots
> tiff(filename="scatter_OligoEset.tif")
> pairs(exprs(OligoEset)[,1:7], pch=".",main="Scatter plots",col=TRUE)
> dev.off()

> ##################################################################################
> #annotation
> ##################################################################################

> # Which platform?
> OligoEset@annotation
> ls("package:mogene20sttranscriptcluster.db")
> ls("package:mogene20stprobeset.db")

> # Get the transcript cluster IDs from the expressionset
> ID <- featureNames(OligoEset)

> # Look up the Gene Symbol, name, and Ensembl Gene ID for each of those IDs
> Symbol <- getSYMBOL(ID, "mogene20sttranscriptcluster.db")
```

```
> Name <- as.character(lookUp(ID, "mogene20sttranscriptcluster.db", "GENENAME"))
> Ensembl <- as.character(lookUp(ID, "mogene20sttranscriptcluster.db", "ENSEMBL"))
> Entrez <- as.character(lookUp(ID, "mogene20sttranscriptcluster.db", "ENTREZID"))
> #GO<-as.character(lookUp(ID, "mogene20sttranscriptcluster.db", "GO"))

> # Make a temporary data frame with all those identifiers
> tmp <- data.frame(ID=ID, Symbol=Symbol, Name=Name, Ensembl=Ensembl,Entrez=Entrez, stringsAsFactors=F)

> # set the featureData for your expressionset using the data frame you created above.
> fData(OligoEset) <- tmp

> ################################################################################
> #Non-specific filtering (GeneFilter)
> ################################################################################

> #intensity filter - (intensity of a gene should be >log2(100) in at least 20% of the samples)

> f1<-pOverA(0.25, log2(100))

> #variance filter - (the interquartile range of log2-intensities should be at least 0.5)
> f2<-function(x)(IQR(x) > 0.5)

> ff<-filterfun(f1,f2)
> selected <- genefilter(OligoEset, ff)
> sum(selected)
> esetSub=OligoEset[selected,]

> ################################################################################
> #differential expression
> ################################################################################
> #limma for linear model of data

> # Export all affy expression values to a tab delimited text file
> write.exprs(esetSub, file="OligoEset.txt")

> #differential expression analysis
> # Create appropriate design matrix and assign column names
> design <- model.matrix(~-1+factor(c("EGG","EGG","EGG","ILSDV","ILSDV","ILSDV","MVA","MVA",
  "MVA","FWPV","FWPV","FWPV", "CNPV","CNPV","CNPV","FPK2", "FPK2", "FPK2", "PEPV","PEPV","PEPV")))
> colnames(design)<-c("EGG","ILSDV","MVA","FWPV","CNPV","FPK2","PEPV")

> # Create appropriate contrast matrix for pairwise comparisons
> contrast.matrix <- makeContrasts(EGG-ILSDV, EGG-MVA, EGG-FWPV, EGG-CNPV, EGG-FPK2,EGG-PEPV,
  levels=design)

> # Fit a linear model for each gene based on the given series of arrays
> fit <- lmFit(esetSub, design)

> # Compute estimated coefficients and standard errors for a given set of
> contrasts
> fit2 <- contrasts.fit(fit, contrast.matrix)

> # Compute moderated t-statistics and log-odds of differential expression by empirical Bayes shrinkage of the
  standard errors towards a common value
> fit2 <- eBayes(fit2)

> # Generate list of top 10 DEGs for first comparison
> topTable(fit2, coef=1, adjust="fdr", sort.by="B",p.value=0.05,number=10)

> #Generate heatmap
> list=c()
> for (i in 1:6){
> genes<-topTable(fit2, coef=i, adjust="fdr", sort.by="B",p.value=0.05,number=600)$ID
> list<-c(list,genes)
> }
> list2<-unique(list)
> length(list2)

> gr <- decideTests(fit2 , adjust.method="none")
> gr2 <- decideTests(fit2 , adjust.method="fdr")

> results<-exprs(OligoEset)[list2,]
```

```
>   colnames(results)<-pd$label

>   heatmap(results,)
>   heatmap(results,scale="none")

>   #heatmap.2 using gplots
>   heatmap.2(results, col=redgreen(75), scale="none", key=TRUE, symkey=FALSE, density.info="none", trace="none",
    cexRow=0.5)

>   #generate gene lists (dif. expr. genes) for each comparison (1-6)
>   listcom1<-topTable(fit2, coef=1, adjust="fdr", sort.by="B",resort.by="logFC", p.value=0.05,number=Inf)
>   write.csv(listcom1, file="listcom1.csv")

>   listcom2<-topTable(fit2, coef=2, adjust="fdr", sort.by="B",resort.by="logFC", p.value=0.05,number=Inf)
>   write.csv(listcom2, file="listcom2.csv")

>   listcom3<-topTable(fit2, coef=3, adjust="fdr", sort.by="B",resort.by="logFC", p.value=0.05,number=Inf)
>   write.csv(listcom3, file="listcom3.csv")

>   listcom4<-topTable(fit2, coef=4, adjust="fdr", sort.by="B",resort.by="logFC", p.value=0.05,number=Inf)
>   write.csv(listcom4, file="listcom4.csv")

>   listcom5<-topTable(fit2, coef=5, adjust="fdr", sort.by="B",resort.by="logFC", p.value=0.05,number=Inf)
>   write.csv(listcom5, file="listcom5.csv")

>   listcom6<-topTable(fit2, coef=6, adjust="fdr", sort.by="B",resort.by="logFC", p.value=0.05,number=Inf)
>   write.csv(listcom6, file="listcom6.csv")


>   ##########################################################################################
>   #analysis with cluster profiler

>   library(DOSE)
>   library(clusterProfiler)
>   library(org.Mm.eg.db)
>   library(GO.db)
>   library(pathview)

>   #comparison1 (egg vs iLSDV)

>   #data input
>   listcom1<-read.csv("c:/R/data/RawData/listcom1.csv")
>   head(listcom1)

>   # annotate with entrez info
>   all_genes1 <- listcom1$Entrez
>   all_genes1 <- all_genes1[!is.na(all_genes1)]
>   all_genes1<-all_genes1[!duplicated(all_genes1)]
>   all_genes1
>   LSDV<-as.character(all_genes1)

>   #GO classification
>   ggo <-groupGO(LSDV, organism="mouse", ont = "BP", level=3, readable = TRUE)
>   head(summary(ggo))

>   #GO enrichment analysis - HYPERGEOMETRIC MODEL (left out universe="")
>   ego<-enrichGO(gene=LSDV,organism="mouse", ont="BP", pvalueCutoff=0.05, readable=TRUE)
>   head(summary(ego))

>   #GO enrichment analysis - KEGG pathway enrichment analysis
>   kk<-enrichKEGG(gene=LSDV,organism="mouse", pvalueCutoff=0.05, readable=TRUE)
>   head(summary(kk))

>   #visualization of results
>   barplot(ggo, drop=TRUE, showCategory=12)
>   barplot(ego, drop=TRUE, showCategory=10)
>   barplot(kk, drop=TRUE, showCategory=10)
>   cnetplot(ego, categorySize="pvalue", foldChange=all_genes1)
>   cnetplot(kk, categorySize="geneNum", foldChange=all_genes1)

>   # visualisation of pathways with pathview
>   require(pathview)
```

```
> setwd("c:/R/data/pathways/lsdv")
> head(summary(kk))

> For each pathway:
> #Type I diabetes mellitus
> mmu04940<-pathview(gene.data=LSDV, pathway.id="mmu04940", species = "mmu", low = list(gene = "green"), mid
  =  list(gene = "gray"), high = list(gene = "red"))

> ##############################################################################################
> #comparison2 (egg vs MVA)

> #data input
> listcom2<-read.csv("c:/R/data/RawData/listcom2.csv")
> head(listcom2)

> # annotate with entrez info
> all_genes2 <- listcom2$Entrez
> all_genes2 <- all_genes2[!is.na(all_genes2)]
> all_genes2<-all_genes2[!duplicated(all_genes2)]
> all_genes2
> MVA<-as.character(all_genes2)

> #GO classification
> ggo2 <-groupGO(MVA, organism="mouse", ont = "BP", level=3, readable = TRUE)
> head(summary(ggo2))

> #GO enrichment analysis - HYPERGEOMETRIC MODEL (left out universe="")
> ego2<-enrichGO(gene=MVA,organism="mouse", ont="BP", pvalueCutoff=0.05, readable=TRUE)
> head(summary(ego2))

> #GO enrichment analysis - KEGG pathway enrichment analysis
> kk2<-enrichKEGG(gene=MVA,organism="mouse", pvalueCutoff=0.05, readable=TRUE)
> head(summary(kk2))

> #visualization of results
> barplot(ggo2, drop=TRUE, showCategory=12)
> barplot(ego2, drop=TRUE, showCategory=10)
> barplot(kk2, drop=TRUE, showCategory=10)
> cnetplot(ego2, categorySize="pvalue", foldChange=all_genes1)
> cnetplot(kk2, categorySize="geneNum", foldChange=all_genes1)

> # visualisation of pathways with pathview
> require(pathview)

> ##############################################################################################
> #comparison3 (egg vs FWPV)

> #data input
> listcom3<-read.csv("c:/R/data/RawData/listcom3.csv")
> head(listcom3)

> # annotate with entrez info
> all_genes3 <- listcom3$Entrez
> all_genes3 <- all_genes3[!is.na(all_genes3)]
> all_genes3<-all_genes3[!duplicated(all_genes3)]
> all_genes3
> FWPV<-as.character(all_genes3)

> #GO classification
> ggo3 <-groupGO(FWPV, organism="mouse", ont = "BP", level=3, readable = TRUE)
> head(summary(ggo3))

> #GO enrichment analysis - HYPERGEOMETRIC MODEL (left out universe="")
> ego3 <-enrichGO(gene=FWPV,organism="mouse", ont="BP", pvalueCutoff=0.05, readable=TRUE)
> head(summary(ego3))

> #GO enrichment analysis - KEGG pathway enrichment analysis
> kk3 <-enrichKEGG(gene=FWPV,organism="mouse", pvalueCutoff=0.05, readable=TRUE)
> head(summary(kk3))

> #visualization of results
> barplot(ggo3, drop=TRUE, showCategory=12)
```

```
>   barplot(ego3, drop=TRUE, showCategory=10)
>   barplot(kk3, drop=TRUE, showCategory=10)
>   cnetplot(ego3, categorySize="pvalue", foldChange=all_genes1)
>   cnetplot(kk3, categorySize="geneNum", foldChange=all_genes1)

>   # visualisation of pathways with pathview
>   require(pathview)

>   ####################################################################################################
>   #comparison4 (egg vs CNPV)

>   #data input
>   listcom4<-read.csv("c:/R/data/RawData/listcom4.csv")
>   head(listcom4)

>   # annotate with entrez info
>   all_genes4 <- listcom4$Entrez
>   all_genes4 <- all_genes4[!is.na(all_genes4)]
>   all_genes4<-all_genes4[!duplicated(all_genes4)]
>   all_genes4
>   CNPV<-as.character(all_genes4)

>   #GO classification
>   ggo4 <-groupGO(CNPV, organism="mouse", ont = "BP", level=3, readable = TRUE)
>   head(summary(ggo4))

>   #GO enrichment analysis - HYPERGEOMETRIC MODEL (left out universe="")
>   ego4<-enrichGO(gene=CNPV,organism="mouse", ont="BP", pvalueCutoff=0.05, readable=TRUE)
>   head(summary(ego4))

>   #GO enrichment analysis - KEGG pathway enrichment analysis
>   kk4<-enrichKEGG(gene=CNPV,organism="mouse", pvalueCutoff=0.05, readable=TRUE)
>   head(summary(kk4))

>   #visualization of results
>   barplot(ggo4, drop=TRUE, showCategory=12)
>   barplot(ego4, drop=TRUE, showCategory=10)
>   barplot(kk4, drop=TRUE, showCategory=10)
>   cnetplot(ego4, categorySize="pvalue", foldChange=all_genes1)
>   cnetplot(kk4, categorySize="geneNum", foldChange=all_genes1)

>   # visualisation of pathways with pathview
>   require(pathview)

>   ####################################################################################################
>   #comparison5 (egg vs FeP2)

>   #data input
>   listcom5<-read.csv("c:/R/data/RawData/listcom5.csv")
>   head(listcom5)

>   # annotate with entrez info
>   all_genes5 <- listcom5$Entrez
>   all_genes5 <- all_genes5[!is.na(all_genes5)]
>   all_genes5<-all_genes5[!duplicated(all_genes5)]
>   all_genes5
>   FeP2<-as.character(all_genes5)

>   #GO classification
>   ggo5 <-groupGO(FeP2, organism="mouse", ont = "BP", level=3, readable = TRUE)
>   head(summary(ggo5))

>   #GO enrichment analysis - HYPERGEOMETRIC MODEL (left out universe="")
>   ego5<-enrichGO(gene=FeP2,organism="mouse", ont="BP", pvalueCutoff=0.05, readable=TRUE)
>   head(summary(ego5))

>   #GO enrichment analysis - KEGG pathway enrichment analysis
>   kk5<-enrichKEGG(gene=FeP2,organism="mouse", pvalueCutoff=0.05, readable=TRUE)
>   head(summary(kk5))

>   #visualization of results
>   barplot(ggo5, drop=TRUE, showCategory=12)
```

```
> barplot(ego5, drop=TRUE, showCategory=10)
> barplot(kk5, drop=TRUE, showCategory=10)
> cnetplot(ego5, categorySize="pvalue", foldChange=all_genes1)
> cnetplot(kk5, categorySize="geneNum", foldChange=all_genes1)

> # visualisation of pathways with pathview
> require(pathview)

> ############################################################################################
> #comparison6 (egg vs PEPV)

> #data input
> listcom6<-read.csv("c:/R/data/RawData/listcom6.csv")
> head(listcom6)

> # annotate with entrez info
> all_genes6 <- listcom6$Entrez
> all_genes6 <- all_genes6[!is.na(all_genes6)]
> all_genes6<-all_genes6[!duplicated(all_genes6)]
> all_genes6
> PEPV<-as.character(all_genes6)

> #GO classification
> ggo6 <-groupGO(PEPV, organism="mouse", ont = "BP", level=3, readable = TRUE)
> head(summary(ggo6))

> #GO enrichment analysis - HYPERGEOMETRIC MODEL (left out universe="")
> ego6<-enrichGO(gene=PEPV,organism="mouse", ont="BP", pvalueCutoff=0.05, readable=TRUE)
> head(summary(ego6))

> #GO enrichment analysis - KEGG pathway enrichment analysis
> kk6<-enrichKEGG(gene=PEPV,organism="mouse", pvalueCutoff=0.05, readable=TRUE)
> head(summary(kk6))

> #visualization of results
> barplot(ggo6, drop=TRUE, showCategory=12)
> barplot(ego6, drop=TRUE, showCategory=10)
> barplot(kk6, drop=TRUE, showCategory=10)
> cnetplot(ego6, categorySize="pvalue", foldChange=all_genes1)
> cnetplot(kk6, categorySize="geneNum", foldChange=all_genes1)


> ############################################################################################
> #Biological Theme Comparison

> allcomparison<-cbind(LSDV,MVA,FWPV,CNPV,FeP2,PEPV)
> allcomparison
> write.csv(allcomparison, file="c:/R/data/RawData/allc.csv")

> allc<-read.csv("c:/R/data/RawData/allc.csv") #removed first column of numbers 1-.. in excel
> names(allc)
> allc[1]

> ck<-compareCluster(geneCluster=allc, fun="enrichKEGG", organism="mouse", pvalueCutoff=0.05, readable=TRUE)
> head(summary(ck))
> cGO<-compareCluster(geneCluster=allc, fun="enrichGO",ont="BP", organism="mouse", pvalueCutoff=0.05,
  readable=TRUE)
> cGOMF<-compareCluster(geneCluster=allc, fun="enrichGO",ont="MF", organism="mouse", pvalueCutoff=0.05,
  readable=TRUE)
> cGOCC<-compareCluster(geneCluster=allc, fun="enrichGO",ont="CC", organism="mouse", pvalueCutoff=0.05,
  readable=TRUE)

> plot(ck, type = "dot", showCategory=NULL, by = "count", title = "KEGG enrichment", font.size = 12)
> plot(ck, type = "bar", showCategory=NULL, by = "count", title = "KEGG enrichment", font.size = 12)
> plot(ck, type = "bar", showCategory=NULL, by = "percentage", title = "KEGG enrichment", font.size = 12)

> plot(cGO, type = "dot", showCategory=30, by = "count", title = "GO enrichment", font.size = 12)
> plot(cGO, type = "bar", showCategory=30, by = "count", title = "GO enrichment", font.size = 12)

> plot(cGOMF, type = "dot", showCategory=20, by = "count", title = "GO enrichment", font.size = 12)
> plot(cGOCC, type = "dot", showCategory=20, by = "count", title = "GO enrichment", font.size = 12)
```

Supplementary File 2

Full list of up regulated genes in mouse spleens in response to modified vaccinia Ankara (MVA), lumpy skin disease virus (LSDV), canarypox virus (CNPV), fowlpox virus (FWPV), pigeonpox (FeP2) and penguinpox virus (PEPV).

Table a1. Full list of annotated up regulated genes in mouse spleens in response to CNPV, FeP2, FWPV, LSDV, MVA and PEPV. Differences in Log_2 Fold Changes (between each virus and the control) are depicted.

Symbol	Name	Entrez	MVA	LSDV	CNPV	FWPV	PEPV	FeP2
**1500012F01Rik**	RIKEN cDNA 1500012F01 gene	68949	1.2	1.1	1.5	1.6	-	-
**1500012F01Rik**	RIKEN cDNA 1500012F01 gene	68949	-	1	-	-	-	-
**2610524H06Rik**	RIKEN cDNA 2610524H06 gene	330173	-	-	1.1	1.2	-	-
**3110062M04Rik**	RIKEN cDNA 3110062M04 gene	78412	1.2	1.7	1.5	1.4	-	-
**3830406C13Rik**	RIKEN cDNA 3830406C13 gene	218734	-	-	1	-	-	-
**4933412E12Rik**	RIKEN cDNA 4933412E12 gene	71086	-	1	-	1	-	-
**9330175E14Rik**	RIKEN cDNA 9330175E14 gene	320377	-	1.1	-	1.1	-	-
**A530040E14Rik**	RIKEN cDNA A530040E14 gene	621875	1.1	-	-	-	-	-
**A530064D06Rik**	RIKEN cDNA A530064D06 gene	328830	2	1.8	2.2	1.9	1.2	-
**Abracl**	ABRA C-terminal like	73112	-	-	-	1	-	-
**Abtb2**	ankyrin repeat and BTB (POZ) domain containing 2	99382	-	1.1	-	1.1	-	-
**Adamts1**	a disintegrin-like and metallopeptidase (reprolysin type) with thrombospondin type 1 motif, 1	11504	-	1.1	-	-	-	-
**Adar**	adenosine deaminase, RNA-specific	56417	-	1	-	-	-	-
**AI607873**	expressed sequence AI607873	226691	1.8	2	1.8	1.6	1.1	-
**Aif1**	allograft inflammatory factor 1	11629	-	-	-	1.1	-	-
**Akr1b10**	aldo-keto reductase family 1, member B10 (aldose reductase)	67861	-	-	1.1	1.1	-	-
**Alox12**	arachidonate 12-lipoxygenase	11684	-	-	1	1.2	-	-
**Alpl**	alkaline phosphatase, liver/bone/kidney	11647	1.3	1.3	1	1.2	-	-
**Amica1**	adhesion molecule, interacts with CXADR antigen 1	270152	-	1.1	-	1.1	-	-
**Angptl4**	angiopoietin-like 4	57875	1.1	1.5	-	1.6	1.1	-
**Anxa1**	annexin A1	16952	-	-	1.6	1.8	-	-
**Anxa2**	annexin A2	12306	-	-	-	1.1	-	-
**Anxa4**	annexin A4	11746	1.8	1.9	1.7	2.1	1.1	-
**Aoah**	acyloxyacyl hydrolase	27052	-	-	1.1	-	-	-
**Apobec1**	apolipoprotein B mRNA editing enzyme, catalytic polypeptide 1	11810	-	-	1.2	1.2	-	-
**Apod**	apolipoprotein D	11815	1.5	1.3	1.5	1.4	-	-
**Apol9b**	apolipoprotein L 9b	71898	2.3	2.7	2.6	2.4	-	-
**Asb13**	ankyrin repeat and SOCS box-containing 13	142688	1.2	1	1.1	1.3	-	-
**Asprv1**	aspartic peptidase, retroviral-like 1	67855	1.2	1.1	1.2	1.3	-	-
**Atf3**	activating transcription factor 3	11910	1.3	1.4	1.2	1.4	1.1	-
**Atp10a**	ATPase, class V, type 10A	11982	1.1	1.2	-	1.1	-	-
**Atp8b1**	ATPase, class I, type 8B, member 1	54670	1	1.3	1	1.3	-	-
**Atp8b4**	ATPase, class I, type 8B, member 4	241633	-	1.4	-	-	-	-
**AW011738**	expressed sequence AW011738	100382	1.1	1.7	1	1	-	-
**AW112010**	expressed sequence AW112010	107350	2	1.8	1.4	1.8	-	-
**B430306N03Rik**	RIKEN cDNA B430306N03 gene	320148	1.2	1.4	1.3	1.5	-	-
**B4galt4**	UDP-Gal:betaGlcNAc beta 1,4-galactosyltransferase, polypeptide 4	56375	-	1.1	-	-	-	-
**B4galt5**	UDP-Gal:betaGlcNAc beta 1,4-galactosyltransferase, polypeptide 5	56336	-	1	-	-	-	-
**BC023105**	cDNA sequence BC023105	667597	1.3	1.5	1.3	1.4	1.1	-
**BC094916**	cDNA sequence BC094916	545384	1.2	1.8	-	-	-	-
**BC147527**	cDNA sequence BC147527	625360	1.2	1.5	-	-	-	-
**Bst2**	bone marrow stromal cell antigen 2	69550	2.3	3	2.1	2.2	-	-
**C1qa**	complement component 1, q subcomponent, alpha polypeptide	12259	-	1.1	1	-	-	-
**C2**	complement component 2 (within H-2S)	12263	1.5	1.8	1.7	1.6	1	-
**Cacnb3**	calcium channel, voltage-dependent, beta 3 subunit	12297	-	1.2	-	1.1	-	-
**Car13**	carbonic anhydrase 13	71934	1	1.2	1.1	1.1	-	-
**Casp1**	caspase 1	12362	1.1	-	1.2	1.1	-	-
**Casp4**	caspase 4, apoptosis-related cysteine peptidase	12363	1.9	1.6	1.9	2	-	-
**Ccdc53**	coiled-coil domain containing 53	67282	-	-	-	1	-	-
**Ccl2**	chemokine (C-C motif) ligand 2	20296	3.5	3.3	2.9	3.3	2.8	-
**Ccl3**	chemokine (C-C motif) ligand 3	20302	2	2.1	2.3	2	1.4	-
**Ccl6**	chemokine (C-C motif) ligand 6	20305	-	-	1.3	1.3	-	-
**Ccl7**	chemokine (C-C motif) ligand 7	20306	3	2.9	2.6	2.9	2.7	1.5
**Ccr5**	chemokine (C-C motif) receptor 5	12774	1.1	1.5	1.3	1.3	-	-
**Ccrl2**	chemokine (C-C motif) receptor-like 2	54199	-	1.5	1.2	1.4	-	-

**Cd274**	CD274 antigen	60533	1.9	2.3	1.6	2.1	1.2	-
**Cd5l**	CD5 antigen-like	11801	1.2	1.5	1.4	1.1	-	-
**Cd69**	CD69 antigen	12515	1.8	1.8	1.5	1.7	-	-
**Cdkn1a**	cyclin-dependent kinase inhibitor 1A (P21)	12575	1.7	2	1.5	2	1.2	-
**Cebpb**	CCAAT/enhancer binding protein (C/EBP), beta	12608	-	1	-	-	-	-
**Cfb**	complement factor B	14962	2.2	2.6	1.6	2	1	-
**Chi3l3**	chitinase 3-like 3	12655	-	1.2	1.4	1.6	-	-
**Chic1**	cysteine-rich hydrophobic domain 1	12212	1.1	1.4	1	-	-	-
**Clec4a2**	C-type lectin domain family 4, member a2	26888	1.2	-	1.5	1.1	-	-
**Cmpk2**	cytidine monophosphate (UMP-CMP) kinase 2, mitochondrial	22169	1.3	2	1.3	1.3	-	-
**Cndp2**	CNDP dipeptidase 2 (metallopeptidase M20 family)	66054	1.2	1.4	1.1	1.4	-	-
**Col4a2**	collagen, type IV, alpha 2	12827	-	1.1	-	-	-	-
**Cp**	ceruloplasmin	12870	1.6	1.7	1.6	1.8	1.2	-
**Csf2rb2**	colony stimulating factor 2 receptor, beta 2, low-affinity (granulocyte-macrophage)	12984	-	1.5	-	1.7	-	-
**Csprs**	component of Sp100-rs	114564	1.3	-	-	-	-	-
**Csprs**	component of Sp100-rs	114564	1.5	1.2	-	-	-	-
**Csprs**	component of Sp100-rs	114564	1.5	1.2	-	-	-	-
**Cst7**	cystatin F (leukocystatin)	13011	1.2	1.4	1.1	1.4	-	-
**Cstb**	cystatin B	13014	1.4	1	1.5	1.5	-	-
**Ctla2a**	cytotoxic T lymphocyte-associated protein 2 alpha	13024	1.1	1	1.1	1.3	-	1.4
**Ctsc**	cathepsin C	13032	1.1	1.1	-	1.1	-	-
**Ctsl**	cathepsin L	13039	-	-	1.1	-	-	-
**Cxcl10**	chemokine (C-X-C motif) ligand 10	15945	2.7	3	2.2	2.8	1.7	-
**Cxcl11**	chemokine (C-X-C motif) ligand 11	56066	4.5	4.4	3.4	4.3	1.5	-
**Cxcl9**	chemokine (C-X-C motif) ligand 9	17329	2.1	2.3	-	1.8	-	-
**Cycs**	cytochrome c, somatic	13063	-	1.1	-	1.1	-	-
**Cycs**	cytochrome c, somatic	13063	-	1.1	-	1.1	-	-
**Cycs**	cytochrome c, somatic	13063	-	1.2	-	1.2	-	-
**Daxx**	Fas death domain-associated protein	13163	2	2.3	1.7	1.9	-	-
**Dcn**	decorin	13179	-	-	-	1.5	-	-
**Ddit4**	DNA-damage-inducible transcript 4	74747	1.2	1.2	1.8	1.3	1.4	-
**Ddx18**	DEAD (Asp-Glu-Ala-Asp) box polypeptide 18	66942	-	-	-	1.1	-	-
**Ddx58**	DEAD (Asp-Glu-Ala-Asp) box polypeptide 58	230073	-	1.3	-	-	-	-
**Ddx60**	DEAD (Asp-Glu-Ala-Asp) box polypeptide 60	234311	1.7	2.6	1.5	1.4	-	-
**Dhx58**	DEXH (Asp-Glu-X-His) box polypeptide 58	80861	1.7	2.5	1.6	1.5	-	-
**Dram1**	DNA-damage regulated autophagy modulator 1	71712	1.2	1.4	-	1.3	-	-
**Dtx3l**	deltex 3-like (Drosophila)	209200	1	1.5	-	-	-	-
**Dusp28**	dual specificity phosphatase 28	67446	-	1	-	-	-	-
**Ear2**	eosinophil-associated, ribonuclease A family, member 2	13587	-	-	1.3	1.5	-	-
**Eif2ak2**	eukaryotic translation initiation factor 2-alpha kinase 2	19106	1.1	2.1	1.1	1.1	-	-
**Emp1**	epithelial membrane protein 1	13730	-	-	-	1.1	-	-
**Epsti1**	epithelial stromal interaction 1 (breast)	108670	-	1.4	-	-	-	-
**F830016B08Rik**	RIKEN cDNA F830016B08 gene	240328	1.5	2.5	1.9	1.9	1.2	-
**Fam136a**	family with sequence similarity 136, member A	66488	-	1.1	-	1.3	-	-
**Fam26f**	family with sequence similarity 26, member F	215900	1.5	1.9	1.1	1.8	-	-
**Fbxw17**	F-box and WD-40 domain protein 17	109082	-	1.2	-	1	-	-
**Fcgr1**	Fc receptor, IgG, high affinity I	14129	2.4	2.6	2.4	2.6	1.4	-
**Fcgr4**	Fc receptor, IgG, low affinity IV	246256	2.5	3.6	2.8	2.9	1.8	-
**Fgl2**	fibrinogen-like protein 2	14190	-	-	-	1.2	-	-
**Folh1**	folate hydrolase 1	53320	3.1	2.7	3	2.8	2.1	1.9
**Fpr1**	formyl peptide receptor 1	14293	1.3	1.1	1.4	1.4	-	-
**Fpr2**	formyl peptide receptor 2	14289	1.8	1.5	1.7	1.9	-	-
**Fscn1**	fascin homolog 1, actin bundling protein (Strongylocentrotus purpuratus)	14086	1.2	1.6	-	1	-	-
**G530011O06Rik**	RIKEN cDNA G530011O06 gene	654820	2.5	1.7	1.8	-	-	-
**Gadd45b**	growth arrest and DNA-damage-inducible 45 beta	17873	1.3	1.5	1.2	1.5	-	-
**Gatm**	glycine amidinotransferase (L-arginine:glycine amidinotransferase)	67092	1.2	1.3	1.3	1.4	-	-
**Gbp1**	guanylate binding protein 1	14468	2.1	2.7	1.6	2.5	-	-
**Gbp10**	guanylate-binding protein 10	626578	2.1	3.2	1.3	1.9	-	-
**Gbp11**	guanylate binding protein 11	634650	3.9	4.5	2.9	4.2	1.6	-
**Gbp2**	guanylate binding protein 2	14469	2.3	2.8	1.5	2.7	-	-
**Gbp3**	guanylate binding protein 3	55932	1.6	1.8	1.2	1.6	-	-
**Gbp4**	guanylate binding protein 4	17472	2.1	2.7	1.3	2.4	-	-
**Gbp5**	guanylate binding protein 5	229898	2	2.8	1.3	2.2	1.1	-
**Gbp7**	guanylate binding protein 7	229900	1.6	2.2	1.2	1.7	-	-
**Gbp8**	guanylate-binding protein 8	76074	1.3	1.4	1.4	2.1	-	-
**Gbp9**	guanylate-binding protein 9	236573	1.2	1.9	1	1.3	-	-
**Gca**	grancalcin	227960	1.2	1.3	1.4	1.6	-	-
**Gdap10**	ganglioside-induced differentiation-associated-protein 10	100504486	1	1.6	-	1	-	-
**Gla**	galactosidase, alpha	11605	1.6	1.8	1.6	1.7	1.1	-
**Glipr2**	GLI pathogenesis-related 2	384009	1.4	1.5	1.1	1.5	-	-

**Gm10495**	predicted gene 10495	100504611	-	-	-	1.3	-	-
**Gm11772**	predicted gene 11772	100503912	1.6	2.2	1.7	1.7	-	-
**Gm12185**	predicted gene 12185	620913	2	2.6	1.3	2.1	-	-
**Gm12250**	predicted gene 12250	631323	1.7	2.9	1.1	1.8	-	-
**Gm13157**	predicted gene 13157	100041677	-	-	-	1.1	-	-
**Gm14446**	predicted gene 14446	667373	3.3	3.4	2.3	2.5	-	-
**Gm15056**	predicted gene 15056	100504014	3.8	3.8	3.4	3.7	2.5	1.6
**Gm17757**	GTPase, very large interferon inducible 1 pseudogene	100417829	-	1.4	-	-	-	-
**Gm17757**	GTPase, very large interferon inducible 1 pseudogene	100417829	-	1.5	-	-	-	-
**Gm1966**	predicted gene 1966	434223	1	1.5	-	-	-	-
**Gm19705**	predicted gene, 19705	100503460	1.5	1.4	1.3	1.5	-	-
**Gm19763**	predicted gene, 19763	100503548	-	-	-	1	-	-
**Gm20134**	predicted gene, 20134	100504251	1.7	1.5	1.2	1.3	-	-
**Gm20559**	predicted gene, 20559	330256	-	1.1	-	-	-	-
**Gm4070**	predicted gene 4070	100042856	-	1.5	-	-	-	-
**Gm4841**	predicted gene 4841	225594	1.8	2.1	2	2.1	2	-
**Gm4841**	predicted gene 4841	225594	2.5	3	2.4	2.8	2.3	-
**Gm4951**	predicted gene 4951	240327	1.5	2.7	1.3	1.5	-	-
**Gm5431**	predicted gene 5431	432555	2.6	3.1	2.4	2.3	-	-
**Gm6548**	eukaryotic translation elongation factor 1 alpha 1 pseudogene	625054	-	1.4	-	-	-	-
**Gm6904**	predicted gene 6904	628693	1.4	1.9	-	-	-	-
**Gm7609**	predicted pseudogene 7609	665378	1.4	1.2	-	-	-	-
**Gm7609**	predicted pseudogene 7609	665378	-	1.5	-	-	-	-
**Gm8979**	very large inducible GTPase 1 pseudogene	668108	-	1.1	-	-	-	-
**Gm8989**	very large inducible GTPase 1 pseudogene	668128	-	1.1	-	-	-	-
**Gm8995**	predicted gene 8995	668139	-	1.1	-	-	-	-
**Gmppb**	GDP-mannose pyrophosphorylase B	331026	-	1.1	-	-	-	-
**Gnb4**	guanine nucleotide binding protein (G protein), beta 4	14696	-	1.2	-	-	-	-
**Gng12**	guanine nucleotide binding protein (G protein), gamma 12	14701	-	-	-	1.1	-	-
**Gnl3**	guanine nucleotide binding protein-like 3 (nucleolar)	30877	-	-	-	1.1	-	-
**Gnpnat1**	glucosamine-phosphate N-acetyltransferase 1	54342	1.1	-	-	1	-	-
**Gp49a**	glycoprotein 49 A	14727	2.6	2.4	2.7	2.7	1.9	2
**Gpr128**	G protein-coupled receptor 128	239853	1.2	1.4	-	1.2	-	-
**Grn**	granulin	14824	-	1.1	-	-	-	-
**Gsdmd**	gasdermin D	69146	-	-	-	1.2	-	-
**Gvin1**	GTPase, very large interferon inducible 1	74558	-	1.5	-	-	-	-
**Gzma**	granzyme A	14938	1.8	1.8	2.2	2	-	-
**Gzmb**	granzyme B	14939	3.7	4.7	4.1	4.2	2.4	-
**H2-Q4**	histocompatibility 2, Q region locus 4	15015	1	1.2	-	1.2	-	-
**H2-Q6**	histocompatibility 2, Q region locus 6	110557	1	1	-	1.1	-	-
**H2-T22**	histocompatibility 2, T region locus 22	15039	1	1.3	-	-	-	-
**H2-T23**	histocompatibility 2, T region locus 23	15040	1	1.3	-	1	-	-
**H2-T24**	histocompatibility 2, T region locus 24	15042	-	1.2	-	-	-	-
**Hdc**	histidine decarboxylase	15186	-	1.4	1.2	1.5	-	-
**Helz2**	helicase with zinc finger 2, transcriptional coactivator	229003	1.1	1.5	-	-	-	-
**Herc6**	hect domain and RLD 6	67138	1.3	1.9	1.1	1.1	-	-
**Hk3**	hexokinase 3	212032	1.5	2	1.7	2	1.2	-
**Hmgcs2**	3-hydroxy-3-methylglutaryl-Coenzyme A synthase 2	15360	1.3	1.1	2.1	1.7	1.8	2.2
**Hnrnph2**	heterogeneous nuclear ribonucleoprotein H2	56258	-	-	-	1.1	-	-
**Hp**	haptoglobin	15439	1.2	1.8	1.7	1.9	1.3	1.3
**Hpse**	heparanase	15442	-	1.1	-	-	-	-
**Hsbp1**	heat shock factor binding protein 1	68196	-	-	1.2	1.2	-	-
**Hsh2d**	hematopoietic SH2 domain containing	209488	1.1	1.4	-	-	-	-
**Hspa1b**	heat shock protein 1B	15511	2.3	2.9	2.4	2.5	-	-
**Ifi202b**	interferon activated gene 202B	26388	2.4	2.6	2.1	2.2	-	-
**Ifi203**	interferon activated gene 203	15950	-	1.1	-	-	-	-
**Ifi204**	interferon activated gene 204	15951	3.3	4	3.2	3.9	-	-
**Ifi205**	interferon activated gene 205	226695	1.4	-	1.6	1.8	-	-
**Ifi27l2a**	interferon, alpha-inducible protein 27 like 2A	76933	-	1.9	-	-	-	-
**Ifi35**	interferon-induced protein 35	70110	1.1	1.5	1.1	1.2	-	-
**Ifi44**	interferon-induced protein 44	99899	2	2.5	1.8	1.6	-	-
**Ifi44l**	interferon-induced protein 44 like	15061	2.1	2.9	2	2	-	-
**Ifih1**	interferon induced with helicase C domain 1	71586	1.3	2.1	1.2	1.2	-	-

**Ifit1**	interferon-induced protein with tetratricopeptide repeats 1	15957	2.2	3.3	2	1.7	-	-
**Ifit2**	interferon-induced protein with tetratricopeptide repeats 2	15958	1.8	2.5	1.8	1.7	-	-
**Ifitm3**	interferon induced transmembrane protein 3	66141	-	1.4	-	-	-	-
**Ifitm6**	interferon induced transmembrane protein 6	213002	1.8	1.9	2.4	2.3	1.6	1.5
**Ighg**	Immunoglobulin heavy chain (gamma polypeptide)	380794	-	-	1.5	1.6	-	-
**Ighg3**	Immunoglobulin heavy constant gamma 3	380795	-	-	1.3	1.2	-	-
**Ighm**	immunoglobulin heavy constant mu	16019	-	-	1.1	-	-	-
**Igtp**	interferon gamma induced GTPase	16145	1.4	2.2	1	1.8	-	-
**Iigp1**	interferon inducible GTPase 1	60440	1.7	2.9	1.2	1.9	-	-
**Il12rb1**	interleukin 12 receptor, beta 1	16161	2	2.4	1.7	2.1	1.1	-
**Il12rb2**	interleukin 12 receptor, beta 2	16162	1.2	1.3	1.3	1.3	-	-
**Il15**	interleukin 15	16168	1.1	1.3	1.2	1	-	-
**Il15ra**	interleukin 15 receptor, alpha chain	16169	1.8	1.9	1.5	1.8	1.2	-
**Il18bp**	interleukin 18 binding protein	16068	1.2	1.7	1.3	1.5	-	-
**Il1a**	interleukin 1 alpha	16175	1.9	2	2.2	2.3	1.8	-
**Il1f9**	interleukin 1 family, member 9	215257	1.3	1.6	1.7	1.8	1.2	1
**Il1rn**	interleukin 1 receptor antagonist	16181	1.3	1.1	-	1.2	-	-
**Il2ra**	interleukin 2 receptor, alpha chain	16184	1.1	1.2	-	1.3	1.1	-
**Il33**	interleukin 33	77125	-	1	-	1.1	-	-
**Irf1**	interferon regulatory factor 1	16362	-	1.3	-	1.1	-	-
**Irf7**	interferon regulatory factor 7	54123	1.7	2.9	1.7	1.1	-	-
**Irg1**	immunoresponsive gene 1	16365	2.6	2.5	1.9	2.5	1.6	-
**Irgm1**	immunity-related GTPase family M member 1	15944	1.4	2.3	1.1	1.4	-	-
**Irgm2**	immunity-related GTPase family M member 2	54396	1	1.7	-	1.2	-	-
**Isg15**	ISG15 ubiquitin-like modifier	100038882	1.6	2.2	1.5	1.4	-	-
**Jhdm1d**	jumonji C domain-containing histone demethylase 1 homolog D (S. cerevisiae)	338523	1.1	1.6	1.1	1.1	-	-
**Kcne4**	potassium voltage-gated channel, Isk-related subfamily, gene 4	57814	1.2	1.4	0	1.3	-	-
**Klk1**	kallikrein 1	16612	-	-	1.3	1	-	-
**Klrk1**	killer cell lectin-like receptor subfamily K, member 1	27007	1.5	1.7	1.6	1.5	-	-
**Lap3**	leucine aminopeptidase 3	66988	1.1	1.5	1	1.5	-	-
**Lcn2**	lipocalin 2	16819	1.4	1.6	2.2	2.2	1.4	1.7
**Lgals3bp**	lectin, galactoside-binding, soluble, 3 binding protein	19039	1.2	1.7	1.1	-	-	-
**Lgals3bp**	lectin, galactoside-binding, soluble, 3 binding protein	19039	-	1.5	-	-	-	-
**Lgals9**	lectin, galactose binding, soluble 9	16859	1.2	1.6	1.1	1.2	-	-
**Lgmn**	legumain	19141	1.3	1.2	1.4	1.4	-	-
**Lilrb3**	leukocyte immunoglobulin-like receptor, subfamily B (with TM and ITIM domains), member 3	18733	-	-	1.1	1	-	-
**Lilrb4**	leukocyte immunoglobulin-like receptor, subfamily B, member 4	14728	1.6	1.5	1.7	1.7	1.1	-
**Lipg**	lipase, endothelial	16891	2.4	2.8	2.4	2.5	1.8	1.5
**Lrrc16a**	leucine rich repeat containing 16A	68732	-	1.2	-	-	-	-
**Lrrc61**	leucine rich repeat containing 61	243371	1.8	1.4	1.8	2.1	1.3	-
**Ltv1**	LTV1 homolog (S. cerevisiae)	353258	-	-	-	1.1	-	-
**Ly6a**	lymphocyte antigen 6 complex, locus A	110454	1.7	1.9	1.2	1.3	-	-
**Ly6c1**	lymphocyte antigen 6 complex, locus C1	17067	1.8	2.2	1.9	1.9	-	-
**Ly6c2**	lymphocyte antigen 6 complex, locus C2	100041546	1.3	1.6	1.3	1.1	-	-
**Ly6g**	lymphocyte antigen 6 complex, locus G	546644	-	1.7	2.5	2.2	-	-
**Ly6i**	lymphocyte antigen 6 complex, locus I	57248	-	1	-	-	-	-
**Ly96**	lymphocyte antigen 96	17087	-	-	1.2	-	-	-
**Lyrm2**	LYR motif containing 2	108755	-	-	-	1	-	-
**Marco**	macrophage receptor with collagenous structure	17167	-	-	1	-	1.5	1.5
**Mcee**	methylmalonyl CoA epimerase	73724	-	-	1.1	-	-	-
**Med10**	mediator of RNA polymerase II transcription, subunit 10 homolog (NUT2, S. cerevisiae)	28077	-	-	-	1.1	-	-
**Med11**	mediator of RNA polymerase II transcription, subunit 11 homolog (S. cerevisiae)	66172	-	-	-	1.1	-	-
**Med4**	mediator of RNA polymerase II transcription, subunit 4 homolog (yeast)	67381	-	-	-	1	-	-
**Mid1**	midline 1	17318	1.3	1.4	-	-	-	-
**Mif**	macrophage migration inhibitory factor	17319	-	1.1	-	-	-	-
**Mir1949**	microRNA 1949	100316700	-	1.2	-	-	-	-
**Mitd1**	MIT, microtubule interacting and transport, domain containing 1	69028	1.2	1.4	1.1	1.2	-	-
**Mki67ip**	Mki67 (FHA domain) interacting nucleolar phosphoprotein	67949	-	-	0	1.2	-	-
**Mlkl**	mixed lineage kinase domain-like	74568	2.1	2.7	2	2.3	-	-
**Mmp13**	matrix metallopeptidase 13	17386	2.7	2.6	2.4	2.4	1.5	-
**Mmp19**	matrix metallopeptidase 19	58223	1.9	2	2	2	1.5	1.2

**Mmp25**	matrix metallopeptidase 25	240047	-	1	-	1.1	-	-
**Mmp8**	matrix metallopeptidase 8	17394	2.7	3	3.1	3.4	2.5	2.8
**Mnda**	myeloid cell nuclear differentiation antigen	381308	1.8	1.8	1.6	1.7	-	-
**Mndal**	myeloid nuclear differentiation antigen like	10004046 2	1.1	1.3	1.1	1.2	-	-
**Mov10**	Moloney leukemia virus 10	17454	1.1	1.5	-	-	-	-
**Mreg**	melanoregulin	381269	-	1.1	-	1.1	-	-
**Mrpl42**	mitochondrial ribosomal protein L42	67270	-	-	1.1	1.2	-	-
**Mrpl54**	mitochondrial ribosomal protein L54	66047	-	1	-	-	-	-
**Ms4a4a**	membrane-spanning 4-domains, subfamily A, member 4A	666907	2.6	2.7	2.6	2.5	1.5	1.1
**Ms4a4c**	membrane-spanning 4-domains, subfamily A, member 4C	64380	1.2	1.5	1.2	1	-	-
**Ms4a4d**	membrane-spanning 4-domains, subfamily A, member 4D	66607	1.6	1.5	1.6	1.8	-	-
**Ms4a6c**	membrane-spanning 4-domains, subfamily A, member 6C	73656	1.1	1.1	1.2	1.2	-	-
**Ms4a6d**	membrane-spanning 4-domains, subfamily A, member 6D	68774	3.2	3.5	3.1	3.3	-	-
**Ms4a7**	membrane-spanning 4-domains, subfamily A, member 7	109225	1.7	1.4	2	1.9	1.4	-
**Msr1**	macrophage scavenger receptor 1	20288	2.3	2	2	2.2	1.4	1.1
**Mvp**	major vault protein	78388	-	1.3	-	1	-	-
**Mx1**	myxovirus (influenza virus) resistance 1	17857	3.2	3.9	3	2.8	-	-
**Mx2**	myxovirus (influenza virus) resistance 2	17858	2.7	3.7	2.1	2.2	-	-
**Myc**	myelocytomatosis oncogene	17869	-	1.1	-	-	-	-
**Myd88**	myeloid differentiation primary response gene 88	17874	-	-	-	1.2	-	-
**N4bp1**	NEDD4 binding protein 1	80750	-	1	-	-	-	-
**Naa25**	N(alpha)-acetyltransferase 25, NatB auxiliary subunit	231713	-	1.2	-	1	-	-
**Nampt**	nicotinamide phosphoribosyltransferase	59027	1.7	1.9	1.5	2	-	-
**Nlrc5**	NLR family, CARD domain containing 5	434341	-	1.8	-	-	-	-
**Nmi**	N-myc (and STAT) interactor	64685	1.3	1.6	1.1	1.5	-	-
**Nod1**	nucleotide-binding oligomerization domain containing 1	107607	-	-	-	1	-	-
**Nol8**	nucleolar protein 8	70930	-	-	-	1	-	-
**Nop56**	NOP56 ribonucleoprotein	67134	-	-	-	1.4	-	-
**Nos2**	nitric oxide synthase 2, inducible	18126	-	-	-	1.1	-	-
**Oas1a**	2'-5' oligoadenylate synthetase 1A	246730	1.5	2.7	1.6	1.4	-	-
**Oas1b**	2'-5' oligoadenylate synthetase 1B	23961	-	1.6	-	-	-	-
**Oas1g**	2'-5' oligoadenylate synthetase 1G	23960	2.3	3.9	2.5	2.3	-	-
**Oas2**	2'-5' oligoadenylate synthetase 2	246728	2.1	3.4	2	1.6	-	-
**Oas3**	2'-5' oligoadenylate synthetase 3	246727	1.1	2.6	1.3	1	-	-
**Oasl1**	2'-5' oligoadenylate synthetase-like 1	231655	2.6	3.5	2.4	2.5	-	-
**Oasl2**	2'-5' oligoadenylate synthetase-like 2	23962	2	3.4	2	2	-	-
**Ogfr**	opioid growth factor receptor	72075	-	1.4	-	-	-	-
**Olfr56**	olfactory receptor 56	18356	1.7	2.1	1.2	1.7	-	-
**P2ry14**	purinergic receptor P2Y, G-protein coupled, 14	140795	-	-	1.3	1.1	-	-
**Parp10**	poly (ADP-ribose) polymerase family, member 10	671535	1.2	1.5	-	1.2	-	-
**Parp11**	poly (ADP-ribose) polymerase family, member 11	101187	1	1.4	-	-	-	-
**Parp12**	poly (ADP-ribose) polymerase family, member 12	243771	1.5	2.5	1.3	1.6	-	-
**Parp14**	poly (ADP-ribose) polymerase family, member 14	547253	1.1	1.7	-	1	-	-
**Parp9**	poly (ADP-ribose) polymerase family, member 9	80285	1.3	1.9	1.1	1.2	-	-
**Pdcd5**	programmed cell death 5	56330	-	-	-	1.2	-	-
**Pde7b**	phosphodiesterase 7B	29863	1.8	2.3	2	1.6	-	-
**Pdk4**	pyruvate dehydrogenase kinase, isoenzyme 4	27273	1.5	1.1	1.7	1.4	1.8	1.8
**Pf4**	platelet factor 4	56744	-	-	1.2	1.1	-	-
**Pfkp**	phosphofructokinase, platelet	56421	-	1	-	1.1	-	-
**Phf11a**	PHD finger protein 11A	219131	1.2	1.4	1.1	1.1	-	-
**Phf11b**	PHD finger protein 11B	236451	2	1.8	1.7	1.8	-	-
**Phf11c**	PHD finger protein 11C	628705	2	2.4	1.7	1.7	-	-
**Phf11d**	PHD finger protein 11D	219132	2.4	2.9	2.4	2.4	1.1	-
**Pi4kb**	phosphatidylinositol 4-kinase, catalytic, beta polypeptide	107650	1.4	1.4	1.3	1.2	-	-
**Pkib**	protein kinase inhibitor beta, cAMP dependent, testis specific	18768	1.2	1.4	-	1.2	-	-
**Pla2g4a**	phospholipase A2, group IVA (cytosolic, calcium-dependent)	18783	-	-	-	1.2	-	-
**Plac8**	placenta-specific 8	231507	1.3	1.7	-	1.3	-	-
**Plau**	plasminogen activator, urokinase	18792	1.2	1.1	-	1.1	-	-
**Plin2**	perilipin 2	11520	1.3	1.5	1.4	1.5	1.1	-
**Pno1**	partner of NOB1 homolog (S. cerevisiae)	66249	-	1.1	-	1.1	-	-
**Pnp**	purine-nucleoside phosphorylase	18950	-	1.2	1.2	1.3	-	-
**Pnpt1**	polyribonucleotide nucleotidyltransferase 1	71701	1.1	-	1.1	1.4	-	-
**Pomp**	proteasome maturation protein	66537	-	-	1	-	-	-
**Pop4**	processing of precursor 4, ribonuclease P/MRP family, (S. cerevisiae)	66161	-	-	1	1.2	-	-
**Ppa1**	pyrophosphatase (inorganic) 1	67895	1.3	2.1	-	1.5	-	-
**Pram1**	PML-RAR alpha-regulated adaptor molecule 1	378460	-	-	1.1	1.1	-	-

**Prdx1**	peroxiredoxin 1	18477	1	-	1	1.2	-	-
**Prf1**	perforin 1 (pore forming protein)	18646	1.2	1.4	1.1	1.2	-	-
**Prmt1**	protein arginine N-methyltransferase 1	15469	-	-	-	1	-	-
**Procr**	protein C receptor, endothelial	19124	1	-	-	1	-	-
**Psat1**	phosphoserine aminotransferase 1	107272	1.3	1.4	1	1.3	-	-
**Psma1**	proteasome (prosome, macropain) subunit, alpha type 1	26440	-	-	1.1	1.3	-	-
**Psma7**	proteasome (prosome, macropain) subunit, alpha type 7	26444	-	1.1	-	1.1	-	-
**Psmb10**	proteasome (prosome, macropain) subunit, beta type 10	19171	1.1	1.4	-	1.3	-	-
**Psmb8**	proteasome (prosome, macropain) subunit, beta type 8 (large multifunctional peptidase 7)	16913	-	1.4	-	1.1	-	-
**Psmc6**	proteasome (prosome, macropain) 26S subunit, ATPase, 6	67089	-	-	-	1	-	-
**Psme1**	proteasome (prosome, macropain) 28 subunit, alpha	19186	-	1.1	-	1	-	-
**Ptgs2**	prostaglandin-endoperoxide synthase 2	19225	2.3	2	1.4	2	1.9	-
**Pydc3**	pyrin domain containing 3	100033459	1.9	2.4	1.7	1.5	-	-
**Pydc4**	pyrin domain containing 4	623121	2.9	3.4	2.2	1.9	-	-
**Pyhin1**	pyrin and HIN domain family, member 1	236312	1.7	1.8	1.5	1.4	-	-
**Rasa4**	RAS p21 protein activator 4	54153	-	1.1	1	1	-	-
**Retnlg**	resistin like gamma	245195	1.1	1.3	1.5	1.6	1.4	1.6
**Rgs1**	regulator of G-protein signaling 1	50778	1.7	1.7	1.4	1.5	1.2	-
**Rnf19b**	ring finger protein 19B	75234	-	1.2	-	1.1	-	-
**Rnf213**	ring finger protein 213	672511	1.4	2.2	1	1	-	-
**Rpf2**	ribosome production factor 2 homolog (S. cerevisiae)	67239	-	1.2	-	1.3	-	-
**Rpl23a**	ribosomal protein L23A	268449	-	-	-	1.3	-	-
**Rsl1d1**	ribosomal L1 domain containing 1	66409	-	-	-	1.2	-	-
**Rtp4**	receptor transporter protein 4	67775	1.4	2.2	1.2	1.1	-	-
**Rundc3b**	RUN domain containing 3B	242819	-	1	-	-	-	-
**S100a8**	S100 calcium binding protein A8 (calgranulin A)	20201	-	1.4	1.6	1.9	-	-
**S100a9**	S100 calcium binding protein A9 (calgranulin B)	20202	-	1.2	-	1.2	-	-
**Scarna6**	small Cajal body-specific RNA 6	1.00E+08	-	-	-1.6	-	-	-
**Scimp**	SLP adaptor and CSK interacting membrane protein	327957	1.2	-	1.2	1.5	-	-
**Sco1**	SCO cytochrome oxidase deficient homolog 1 (yeast)	52892	1.5	1.6	1.4	1.6	-	-
**Sepw1**	selenoprotein W, muscle 1	20364	1.1	1.2	1.1	1.1	-	-
**Serpina3f**	serine (or cysteine) peptidase inhibitor, clade A, member 3F	238393	1.9	2.7	1.3	2.2	1.3	-
**Serpinb6b**	serine (or cysteine) peptidase inhibitor, clade B, member 6b	20708	-	-	-	1.2	-	-
**Serpinb9**	serine (or cysteine) peptidase inhibitor, clade B, member 9	20723	1.3	1.1	1.1	1.4	-	-
**Serpinb9b**	serine (or cysteine) peptidase inhibitor, clade B, member 9b	20706	1.2	1	1.1	-	-	-
**Serpine1**	serine (or cysteine) peptidase inhibitor, clade E, member 1	18787	1.1	-	-	1	-	-
**Setdb2**	SET domain, bifurcated 2	239122	1.3	1.8	1.1	1.1	-	-
**Sfrp1**	secreted frizzled-related protein 1	20377	1	1	1.1	1.2	-	-
**Sgcb**	sarcoglycan, beta (dystrophin-associated glycoprotein)	24051	1.7	1.5	1.8	2	-	-
**Slamf8**	SLAM family member 8	74748	-	1.1	-	1.2	-	-
**Slc15a3**	solute carrier family 15, member 3	65221	-	1.3	-	1.1	-	-
**Slc25a22**	solute carrier family 25 (mitochondrial carrier, glutamate), member 22	68267	-	1.2	1	1.2	-	-
**Slfn1**	schlafen 1	20555	1.8	1.9	1.3	1.5	-	-
**Slfn2**	schlafen 2	20556	-	1.1	-	-	-	-
**Slfn3**	schlafen 3	20557	1.3	1.7	1.5	1.7	-	-
**Slfn4**	schlafen 4	20558	1.9	3	2	1.8	-	-
**Slfn5**	schlafen 5	327978	1.5	2.4	1.4	1.1	-	-
**Slfn8**	schlafen 8	276950	1.2	1.5	-	-	-	-
**Slfn9**	schlafen 9	237886	1.5	2.4	1.4	1.7	-	-
**Snora81**	small nucleolar RNA, H/ACA box 81	1.00E+08	-	1.1	-	-	-	-
**Snord12**	small nucleolar RNA, C/D box 12	1.00E+08	-	1.9	-	-	-	-
**Snord52**	small nucleolar RNA, C/D box 52	1.00E+08	-	1.2	-	-	-	-
**Snord72**	small nucleolar RNA, C/D box 72	1.00E+08	-	1.4	-	-	-	-
**Snrpb2**	U2 small nuclear ribonucleoprotein B	20639	-	-	1.1	1	-	-
**Snx10**	sorting nexin 10	71982	-	1.2	-	1.1	-	-
**Snx6**	sorting nexin 6	72183	-	-	1.1	1.3	-	-
**Socs1**	suppressor of cytokine signaling 1	12703	1.6	2.2	-	1.9	-	-
**Socs2**	suppressor of cytokine signaling 2	216233	1	1.4	-	1.8	-	-
**Sp100**	nuclear antigen Sp100	20684	1.1	1.7	1.4	-	-	-
**Sp100**	nuclear antigen Sp100	20684	-	1.4	-	-	-	-
**Sp140**	Sp140 nuclear body protein	434484	-	1.1	-	-	-	-
**Spon1**	spondin 1, (f-spondin) extracellular matrix protein	233744	1.6	1.9	1.5	1.7	-	-
**Stat1**	signal transducer and activator of transcription 1	20846	-	1.4	-	-	-	-
**Stat2**	signal transducer and activator of transcription 2	20847	1.4	1.9	1.2	1.5	-	-
**Stxbp3a**	syntaxin binding protein 3A	20912	1.1	1.1	1.3	1.2	-	-

**Taf1d**	TATA box binding protein (Tbp)-associated factor, RNA polymerase I, D	75316	-	2.4	-	-	-	-
**Tap1**	transporter 1, ATP-binding cassette, sub-family B (MDR/TAP)	21354	-	1.1	-	-	-	-
**Tap2**	transporter 2, ATP-binding cassette, sub-family B (MDR/TAP)	21355	-	1	-	-	-	-
**Tdrd7**	tudor domain containing 7	100121	-	1.3	-	-	-	-
**Tfec**	transcription factor EC	21426	1.6	1	1.7	1.6	-	-
**Tgm2**	transglutaminase 2, C polypeptide	21817	1.3	1.7	1.1	1.5	1	-
**Tgtp1**	T cell specific GTPase 1	21822	-	1.4	-	1.1	-	-
**Tgtp2**	T cell specific GTPase 2	1.00E+08	1.6	2.7	-	1.5	-	-
**Timp1**	tissue inhibitor of metalloproteinase 1	21857	2.8	2.9	2.4	2.9	1.8	1.6
**Tlr13**	toll-like receptor 13	279572	1.4	1	1.7	1.5	1	-
**Tlr3**	toll-like receptor 3	142980	1	1.4	1.2	1.1	-	-
**Tlr7**	toll-like receptor 7	170743	1.1	1.2	1.2	-	-	-
**Tlr8**	toll-like receptor 8	170744	1.1	1.1	1.3	1	-	-
**Tm4sf1**	transmembrane 4 superfamily member 1	17112	-	-	-	1	-	-
**Tmem106a**	transmembrane protein 106A	217203	-	1.1	-	1.1	-	-
**Tmem184b**	transmembrane protein 184b	223693	-	1.1	-	-	-	-
**Tmem67**	transmembrane protein 67	329795	1.3	1.4	1.2	1.4	-	-
**Tnfsf10**	tumor necrosis factor (ligand) superfamily, member 10	22035	2	2.3	2	1.9	-	-
**Tnn**	tenascin N	329278	-	1.2	-	-	-	-
**Tomm70a**	translocase of outer mitochondrial membrane 70 homolog A (yeast)	28185	-	1.1	-	-	-	-
**Tor3a**	torsin family 3, member A	30935	1.6	2.1	1.4	1.4	-	-
**Trafd1**	TRAF type zinc finger domain containing 1	231712	1.1	1.6	1	1.1	-	-
**Trem3**	triggering receptor expressed on myeloid cells 3	58218	1.1	1.4	1.2	1.5	-	-
**Trex1**	three prime repair exonuclease 1	22040	-	1.2	-	1	-	-
**Trim12c**	tripartite motif-containing 12C	319236	-	1.1	-	-	-	-
**Trim12c**	tripartite motif-containing 12C	319236	-	1.5	1.2	1.3	-	-
**Trim21**	tripartite motif-containing 21	20821	1.1	1.3	-	1.4	-	-
**Trim25**	tripartite motif-containing 25	217069	-	1	-	-	-	-
**Trim30a**	tripartite motif-containing 30A	20128	1.3	2.1	-	1.1	-	-
**Trim30c**	tripartite motif-containing 30C	434219	2.6	3.4	2.5	2.2	-	-
**Trim30d**	tripartite motif-containing 30D	209387	3.1	3.5	3.4	2.7	1.4	-
**Trim34b**	tripartite motif-containing 34B	434218	-	1.1	-	-	-	-
**Tspo**	translocator protein	12257	1.1	1.4	1.3	1.3	-	-
**Tubb1**	tubulin, beta 1 class VI	545486	-	-	-	1.3	-	-
**Uba7**	ubiquitin-like modifier activating enzyme 7	74153	-	1.1	-	-	-	-
**Upp1**	uridine phosphorylase 1	22271	-	1.1	-	-	-	-
**Usp18**	ubiquitin specific peptidase 18	24110	2.2	3.1	2	1.8	-	-
**Vwa5a**	von Willebrand factor A domain containing 5A	67776	-	1.3	-	1.2	-	-
**Wars**	tryptophanyl-tRNA synthetase	22375	1.1	1.6	-	1.4	-	-
**Wfdc17**	WAP four-disulfide core domain 17	1.00E+08	2.7	2.4	2.8	2.7	-	-
**Xaf1**	XIAP associated factor 1	327959	1.3	2.1	1.1	1.2	-	-
**Xdh**	xanthine dehydrogenase	22436	2.3	3	2.4	2.4	1.2	-
**Zbp1**	Z-DNA binding protein 1	58203	1.9	2.7	1.3	1.6	-	-
**Zcchc2**	zinc finger, CCHC domain containing 2	227449	-	1.1	-	-	-	-
**Zfp1**	zinc finger protein 1	22640	-	1.1	1.1	1.2	-	-
**Znfx1**	zinc finger, NFX1-type containing 1	98999	1	1.6	1	1.1	-	-
**Znrd1**	zinc ribbon domain containing, 1	66136	-	-	1.1	1.2	-	-
**Zufsp**	zinc finger with UFM1-specific peptidase domain	72580	-	1.5	-	-	-	-

Supplementary File 3

Full list of down regulated genes in mouse spleens in response to modified vaccinia Ankara (MVA), lumpy skin disease virus (LSDV), canarypox virus (CNPV), fowlpox virus (FWPV), pigeonpox (FeP2) and penguinpox virus (PEPV).

Table a2. Full list of annotated down regulated genes in mouse spleens in response to canarypox virus (CNPV), pigeonpox (FeP2), fowlpox virus (FWPV), lumpy skin disease virus (LSDV), modified vaccinia Ankara (MVA) and penguinpox virus (PEPV). Differences in Log_2 Fold Changes (between each virus and the control) are depicted.

Symbol	Name	Entrez	MVA	LSDV	CNPV	FWPV	PEPV	FeP2
**2510003D18Rik**	RIKEN cDNA 2510003D18 gene	72317	-1.1	-1.3	-	-	-	-
**2900052N01Rik**	RIKEN cDNA 2900052N01 gene	73040	-	-1.1	-	-	-	-
**5430401H09Rik**	RIKEN cDNA 5430401H09 gene	100504461	-1.5	-1.1	-	-	-	-
**9430076G02Rik**	RIKEN cDNA 9430076G02 gene	77433	-2.2	-1.8	-1.9	-1.9	-1.5	-1.3
**A530099J19Rik**	RIKEN cDNA A530099J19 gene	319293	-1.3	-1.5	-	-	-	-
**Abca9**	ATP-binding cassette, sub-family A (ABC1), member 9	217262	-1.3	-1.5	-	-1.1	-	-
**Adam23**	a disintegrin and metallopeptidase domain 23	23792	-1.3	-1.3	-1.0	-1.2	-	-
**Adamdec1**	ADAM-like, decysin 1	58860	-	-1.1	-	-	-	-
**Add2**	adducin 2 (beta)	11519	-1.3	-	-1.1	-	-	-
**Ano1**	anoctamin 1, calcium activated chloride channel	101772	-1.4	-1.4	-	-	-	-
**Aplnr**	apelin receptor	23796	-1.4	-1.5	-1.2	-1.2	-	-
**Aqp1**	aquaporin 1	11826	-1.5	-1.2	-1.2	-1.1	-	-
**Aspm**	asp (abnormal spindle)-like, microcephaly associated (Drosophila)	12316	-1.2	-	-	-	-	-
**Atp2b4**	ATPase, Ca++ transporting, plasma membrane 4	381290	-1.4	-	-1.1	-1.1	-	-
**B3gnt8**	UDP-GlcNAc:betaGal beta-1,3-N-acetylglucosaminyltransferase 8	232984	-1.3	-1.3	-1.1	-1.1	-	-
**Btnl10**	butyrophilin-like 10	192194	-1.8	-1.3	-1.2	-1.2	-1.1	-
**Cacna1g**	calcium channel, voltage-dependent, T type, alpha 1G subunit	12291	-1.3	-	-	-	-	-
**Ccdc80**	coiled-coil domain containing 80	67896	-1.4	-1.4	-1.3	-	-1.1	-
**Ccl21a**	chemokine (C-C motif) ligand 21A (serine)	18829	-1.3	-1.7	-	-	-	-
**Ccl21a**	chemokine (C-C motif) ligand 21A (serine)	18829	-1.3	-1.7	-	-	-	-
**Ccl21b**	chemokine (C-C motif) ligand 21B (leucine)	100042493	-1.2	-1.6	-	-	-	-
**Ccl21b**	chemokine (C-C motif) ligand 21B (leucine)	100042493	-1.3	-1.6	-	-	-	-
**Ccl21b**	chemokine (C-C motif) ligand 21B (leucine)	100042493	-1.2	-1.7	-	-	-	-
**Ccl21b**	chemokine (C-C motif) ligand 21B (leucine)	100042493	-1.2	-1.7	-	-	-	-
**Ccl21b**	chemokine (C-C motif) ligand 21B (leucine)	100042493	-1.2	-1.7	-	-	-	-
**Ccl21b**	chemokine (C-C motif) ligand 21B (leucine)	100042493	-1.2	-1.7	-	-	-	-
**Ccl21c**	chemokine (C-C motif) ligand 21C (leucine)	65956	-1.2	-1.6	-	-	-	-
**Cd209a**	CD209a antigen	170786	-1.7	-2.2	-1.1	-1.5	-	-
**Cd209b**	CD209b antigen	69165	-1.2	-1.3	-	-	-	-
**Cd59a**	CD59a antigen	12509	-	-1.2	-	-	-	-
**Cd7**	CD7 antigen	12516	-	-1.1	-	-	-	-
**Cdh8**	cadherin 8	12564	-	-1.0	-	-	-	-
**Cdkl1**	cyclin-dependent kinase-like 1 (CDC2-related kinase)	71091	-1.2	-	-	-	-	-
**Cdkn2c**	cyclin-dependent kinase inhibitor 2C (p18, inhibits CDK4)	12580	-1.3	-	-	-	-	-
**Cep76**	centrosomal protein 76	225659	-1.1	-	-	-	-	-
**Cit**	citron	12704	-1.3	-	-	-	-	-
**Cldn13**	claudin 13	57255	-1.5	-1.2	-1.1	-	-	-
**Col14a1**	collagen, type XIV, alpha 1	12818	-1.6	-1.6	-1.3	-1.4	-1.0	-
**Cpm**	carboxypeptidase M	70574	-1.3	-1.3	-1.1	-1.1	-	-
**Csmd3**	CUB and Sushi multiple domains 3	239420	-1.0	-1.1	-	-	-	-
**Ctsf**	cathepsin F	56464	-1.1	-	-	-	-	-
**Depdc1a**	DEP domain containing 1a	76131	-1.1	-	-	-	-	-
**Diap3**	diaphanous homolog 3 (Drosophila)	56419	-1.1	-	-	-	-	-
**E2f2**	E2F transcription factor 2	242705	-1.5	-	-1.2	-1.2	-	-
**E2f8**	E2F transcription factor 8	108961	-1.4	-1.0	-1.1	-	-	-
**Emr4**	EGF-like module containing, mucin-like, hormone receptor-like sequence 4	52614	-1.4	-1.8	-1.1	-1.5	-1.3	-
**Esm1**	endothelial cell-specific molecule 1	71690	-	-1.1	-	-	-	-
**Fbln5**	fibulin 5	23876	-2.0	-2.0	-1.4	-1.3	-1.1	-
**Fcamr**	Fc receptor, IgA, IgM, high affinity	64435	-1.1	-	-1.5	-	-	-

**Fcer2a**	Fc receptor, IgE, low affinity II, alpha polypeptide	14128	-2.6	-3.1	-2.4	-2.5	-2.2	-1.3
**Fhdc1**	FH2 domain containing 1	229474	-1.42824	-	-	-	-	-
**Fn3k**	fructosamine 3 kinase	63828	-2.0	-1.5	-1.5	-1.5	-1.1	-
**Gas6**	growth arrest specific 6	14456	-	-1.1	-	-	-	-
**Glrx5**	glutaredoxin 5 homolog (S. cerevisiae)	73046	-1.3	-	-1.1	-	-	-
**Gm12839**	cytochrome P450, family 4, subfamily b, polypeptide 1 pseudogene	631037	-1.7	-1.3	-1.1	-1.4	-1.4	-
**Gm20236**	predicted gene, 20236	100504453	-1.9	-1.2	-1.5	-1.6	-1.3	-
**Gm20236**	predicted gene, 20236	100504453	-1.9	-1.2	-1.5	-1.6	-1.3	-
**Gpsm2**	G-protein signalling modulator 2 (AGS3-like, C. elegans)	76123	-1.1	-	-	-	-	-
**H2-M2**	histocompatibility 2, M region locus 2	14990	-1.1	-1.4	-1.0	-1.0	-1.1	-
**Hmmr**	hyaluronan mediated motility receptor (RHAMM)	15366	-1.0	-	-	-	-	-
**Hs3st2**	heparan sulfate (glucosamine) 3-O-sulfotransferase 2	195646	-1.4	-1.3	-1.0	-1.0	-	-
**Ifi27l1**	interferon, alpha-inducible protein 27 like 1	52668	-1.4	-1.3	-	-	-	-
**Igfbp3**	insulin-like growth factor binding protein 3	16009	-	-1.0	-	-	-	-
**Igk**	immunoglobulin kappa chain complex	243469	-	-1.0	-	-	-	-
**Kcnj16**	potassium inwardly-rectifying channel, subfamily J, member 16	16517	-	-1.1	-	-	-	-
**Kel**	Kell blood group	23925	-1.5	-1.5	-	-	-	-
**Kif14**	kinesin family member 14	381293	-1.3	-	-	-	-	-
**Kif23**	kinesin family member 23	71819	-1.1	-	-	-	-	-
**Klhl14**	kelch-like 14	225266	-1.5	-1.6	-1.3	-1.4	-1.2	-
**Kynu**	kynureninase (L-kynurenine hydrolase)	70789	-1.3	-1.4	-1.0	-1.1	-	-
**Lilra5**	leukocyte immunoglobulin-like receptor, subfamily A (with TM domain), member 5	232801	-	-1.1	-	-	-	-
**Limch1**	LIM and calponin homology domains 1	77569	-1.1	-1.2	-	-	-	-
**Lphn3**	latrophilin 3	319387	-	-1.1	-	-	-	-
**Mgll**	monoglyceride lipase	23945	-1.3	-	-	-	-	-
**Mgst3**	microsomal glutathione S-transferase 3	66447	-1.6	-1.0	-	-1.0	-	-
**Mir687**	microRNA 687	751541	-1.4	-	-	-	-	-
**Nusap1**	nucleolar and spindle associated protein 1	108907	-1.1	-	-	-	-	-
**Nxpe4**	neurexophilin and PC-esterase domain family, member 4	244853	-1.2	-	-	-	-	-
**Olfml2a**	olfactomedin-like 2A	241327	-	-1.0	-	-	-	-
**Paqr9**	progestin and adipoQ receptor family member IX	75552	-1.1	-	-	-	-	-
**Pcp4**	Purkinje cell protein 4	18546	-1.2	-1.5	-	-	-	-
**Pcx**	pyruvate carboxylase	18563	-1.4	-	-	-	-	-
**Pkhd1l1**	polycystic kidney and hepatic disease 1-like 1	192190	-2.6	-2.0	-2.1	-2.1	-1.7	-
**Pklr**	pyruvate kinase liver and red blood cell	18770	-1.1	-	-	-	-	-
**Ppox**	protoporphyrinogen oxidase	19044	-1.1	-1.1	-	-	-	-
**Prelp**	proline arginine-rich end leucine-rich repeat	116847	-1.0	-1.2	-	-	-	-
**Prkcg**	protein kinase C, gamma	18752	-	-1.02954	-	-	-	-
**Reep6**	receptor accessory protein 6	70335	-1.1	-	-	-	-	-
**Sec14l2**	SEC14-like 2 (S. cerevisiae)	67815	-1.7	-1.3	-1.5	-1.3	-1.2	-
**Sh3yl1**	Sh3 domain YSC-like 1	24057	-1.6	-1.2	-	-	-	-
**Slc12a2**	solute carrier family 12, member 2	20496	-1.0	-1.0	-	-	-	-
**Slc16a10**	solute carrier family 16 (monocarboxylic acid transporters), member 10	72472	-1.4	-	-	-1.1	-	-
**Slc2a4**	solute carrier family 2 (facilitated glucose transporter), member 4	20528	-1.7	-1.0	-	-1.1	-	-
**Slc38a5**	solute carrier family 38, member 5	209837	-1.4	-1.2	-1.0	-	-	-
**Slc6a20a**	solute carrier family 6 (neurotransmitter transporter), member 20A	102680	-1.4	-1.3	-1.0	-1.0	-	-
**Sned1**	sushi, nidogen and EGF-like domains 1	208777	-1.1	-1.1	0	-1.0	-	-
**Snx22**	sorting nexin 22	382083	-1.4	-1.1	-	-	-	-
**Sox6**	SRY-box containing gene 6	20679	-1.8	-1.2	-1.2	-1.4	-1.1	-
**Sptb**	spectrin beta, erythrocytic	20741	-1.9	-1.4	-1.6	-1.5	-1.3	-1.3
**St8sia6**	ST8 alpha-N-acetyl-neuraminide alpha-2,8-sialyltransferase 6	241230	-	-1.0	-	-	-	-
**Tac2**	tachykinin 2	21334	-1.0	-1.5	-	-	-	-
**Tfrc**	transferrin receptor	22042	-1.2	-	-	-	-	-
**Timd4**	T cell immunoglobulin and mucin domain containing 4	276891	-1.1	-1.3	-	-	-	-
**Tlr11**	toll-like receptor 11	239081	-1.0	-	-	-	-	-
**Tspan33**	tetraspanin 33	232670	-1.6	-	-1.3	-1.1	-	-
**Tspan8**	tetraspanin 8	216350	-1.4	-	-	-1.0	-	-
